# Nitrogen Fertilization and Native C_4_ Grass Species Alter Abundance, Activity, and Diversity of Soil Diazotrophic Communities

**DOI:** 10.3389/fmicb.2021.675693

**Published:** 2021-07-08

**Authors:** Jialin Hu, Jonathan D. Richwine, Patrick D. Keyser, Lidong Li, Fei Yao, Sindhu Jagadamma, Jennifer M. DeBruyn

**Affiliations:** ^1^Department of Biosystems Engineering and Soil Science, University of Tennessee, Knoxville, Knoxville, TN, United States; ^2^Department of Forestry, Wildlife and Fisheries, University of Tennessee, Knoxville, TN, United States; ^3^United States Department of Agriculture—Agricultural Research Service, Agroecosystem Management Research Unit, University of Nebraska-Lincoln, Lincoln, NE, United States

**Keywords:** nitrogen fertilization, switchgrass (*Panicum virgatum* L.), big bluestem (*Andropogon gerardii*), diazotroph community, *nifH* abundance, *nifH* amplicon sequencing

## Abstract

Native C_4_ grasses have become the preferred species for native perennial pastures and bioenergy production due to their high productivity under low soil nitrogen (N) status. One reason for their low N requirement is that C_4_ grasses may benefit from soil diazotrophs and promote biological N fixation. Our objective was to evaluate the impact of N fertilization rates (0, 67, and 202 kg N ha^–1^) and grass species (switchgrass [*Panicum virgatum*] and big bluestem [*Andropogon gerardii*]) on the abundance, activity, diversity, and community composition of soil diazotrophs over three agricultural seasons (grass green-up, initial harvest, and second harvest) in a field experiment in East Tennessee, United States. Nitrogen fertilization rate had a stronger influence on diazotroph population size and activity (determined by *nifH* gene and transcript abundances) and community composition (determined by *nifH* gene amplicon sequencing) than agricultural season or grass species. Excessive fertilization (202 kg N ha^–1^) resulted in fewer *nifH* transcripts compared to moderate fertilization (67 kg N ha^–1^) and decreased both richness and evenness of diazotrophic community, reflecting an inhibitory effect of high N application rates on soil diazotrophic community. Overall, cluster I and cluster III diazotrophs were dominant in this native C_4_ grass system. Diazotroph population size and activity were directly related to soil water content (SWC) based on structural equation modeling. Soil pH, SWC, and C and N availability were related to the variability of diazotrophic community composition. Our results revealed relationships between soil diazotrophic community and associated soil properties, adding to our understanding of the response of soil diazotrophs to N fertilization and grass species in native C_4_ grass systems.

## Introduction

Nitrogen (N) is an essential nutrient for plant growth, and its availability limits the productivity of global ecosystems (Vitousek and Howarth, 1991; LeBauer and Treseder, 2008). Biological N fixation (BNF) is the microbial transformation of atmospheric nitrogen (N_2_) to ammonia (NH_3_), which is carried out by diazotrophs, bacteria and archaea that possess the nitrogenase enzyme complex encoded by the *nif* genes (*nifH*, *nifD*, and *nifK*) (Gaby and Buckley, 2011). BNF is estimated to contribute around 40–100 Tg N input to terrestrial ecosystems per year (Vitousek et al., 2013) and account for about half of annual N inputs into biosphere (Vitousek et al., 1997; Gaby and Buckley, 2014). The distribution of diazotrophs have been investigated in various environments, including seawater (Gilbert et al., 2011; Yang et al., 2019), sediments (Burns et al., 2002; Dang et al., 2013; Andersson et al., 2014), and soils (Feng et al., 2019; Han et al., 2019). Among them, soil harbors the most diverse diazotrophic microbial communities. Diazotrophs belonging to phyla Proteobacteria and Cyanobacteria are typically the most abundant N-fixing bacteria in soil (Gaby and Buckley, 2011). However, soil diazotrophic population size, diversity, and community composition are highly variable across study sites due to different soil characteristics such as pH (Wang Y. et al., 2017), moisture (Srivastava and Ambasht, 1994), organic matter content (Gupta et al., 2014), and temperature (Feng et al., 2019), which are all affected by season (Pereira e Silva et al., 2011; Yeager et al., 2012; Pereira et al., 2013), plant species (Davis et al., 2011), and N input (Deslippe et al., 2005; Wang et al., 2016, Wang C. et al., 2017).

The highly conserved *nifH* gene has been widely used to identify and quantify N-fixing bacteria and archaea (Raymond et al., 2004; Gaby and Buckley, 2012; Gaby et al., 2018; Yang et al., 2019). *nifH* gene abundances are commonly used to estimate population size, while expression or transcript abundances are used as an indicator of activity. The genetic divergence of *nifH* does not correlate well to the divergence of 16S rRNA genes in diazotrophs: it has been reported that species having <3% sequence dissimilarity in their 16S rRNA genes can have up to 23% sequence dissimilarity in *nifH* (Gaby and Buckley, 2014). Based on *nifH* phylogenies, diazotrophs can be classified to five main clusters (clusters I–V). In general, cluster I contains aerobic and facultatively anaerobic diazotrophs belonging to Proteobacteria, Cyanobacteria, Firmicutes, and Actinobacteria. Cluster II contains a relatively small number of diazotrophs belonging to methanogenic archaea. Cluster III are mainly anaerobic bacteria and archaea, including spirochetes, methanogens, acetogens, sulfate-reducing bacteria, Chloracea, and Clostridia (Young, 1992; Zehr et al., 2003; Gaby and Buckley, 2014). Clusters IV and V are *nifH* paralogs that are not involved in N fixation (Zehr et al., 2003). The dominant clusters are highly variable across study sites. For example, cluster I and II diazotrophs were reported to be dominant in the tundra (Feng et al., 2019). Cluster II and III dominated a mangrove ecosystem in South China (Liu et al., 2020), whereas cluster I and III were more prevalent in tropical mangrove ecosystems in Singapore (Jing et al., 2015), forest soils (Yeager et al., 2005; Babur et al., 2014; Izquierdo and Klaus, 2015), cropland soils (Mårtensson et al., 2009) and pasture soils (Babur et al., 2014; Gupta et al., 2019).

Grasslands account for 46.8% of all agricultural lands in the United States (USDA-NASS, 2012). In the humid Southeastern United States, grasslands cover 19.3 million hectares (35.4% of United States agricultural land) (USDA-NASS, 2012). In recent years, the emerging bioenergy economy based on dedicated herbaceous energy crops has promoted the widespread planting of native C_4_ perennial grasses such as switchgrass (*Panicum virgatum* [SG]) and big bluestem (*Andropogon gerardii* [BB]). These native C_4_ grasses are drought-tolerant, highly productive, grow well at low N availability conditions, and ultimately contribute to biodiversity and sustainability of grassland ecosystems (Parrish and Fike, 2005; Fike et al., 2006). One explanation for the low N demands of these native grasses is the high volume of organic matter produced, accumulated, and deposited by the large root systems and/or leaf litter that feeds the growth and activity of diazotrophs in the soil (Parrish and Fike, 2005; Keuter et al., 2014; Gupta et al., 2019). Previous studies have reported that bioenergy grasses promoted diazotrophs (Mao et al., 2013) and root-associated diazotrophic communities contributed to meeting N demands of switchgrass (Smercina et al., 2020). In addition, different grass species may support different diazotrophic communities due to root systems with varying soil C input capacity and N use efficiency. For example, compared to switchgrass, big bluestem-dominated stands exhibited greater soil C accrual after five growing seasons (Adkins et al., 2019) and showed greater N use efficiency (Friesen and Cattani, 2017).

Soil nitrogen content is a key factor affecting BNF (Patra et al., 2007; Smercina et al., 2019). Many studies have reported that highly available N (urea-N, NH_4_^+^-N, and NO_3_^–^-N) suppressed *nifH* abundance and expression (Cusack et al., 2009; Lindsay et al., 2010; Pereira et al., 2013; Reardon et al., 2014; Wang C. et al., 2017). Diazotrophs preferentially use easily available exogenous N as this takes less energy to assimilate compared to fixing atmospheric N, which consequently down-regulates the expression of N-fixation genes (Chapin et al., 2002; Smercina et al., 2019). Moreover, enhanced soil acidification caused by excessive N fertilization can also reduce diazotroph abundance and functional activity, and change diazotrophic community composition and diversity (Limmer and Drake, 1996; Han et al., 2019). In contrast, under low N availability, such as bioenergy cropping systems in marginal lands, diazotroph abundance and functional activity may be promoted because BNF offers diazotrophs a competitive advantage (Lindsay et al., 2010; Babur et al., 2014; Wang C. et al., 2017; Smercina et al., 2019). Therefore, N fertilization is likely to affect the diazotrophic microbial community in C_4_ grassland ecosystems with low N availability.

**FIGURE 1:**
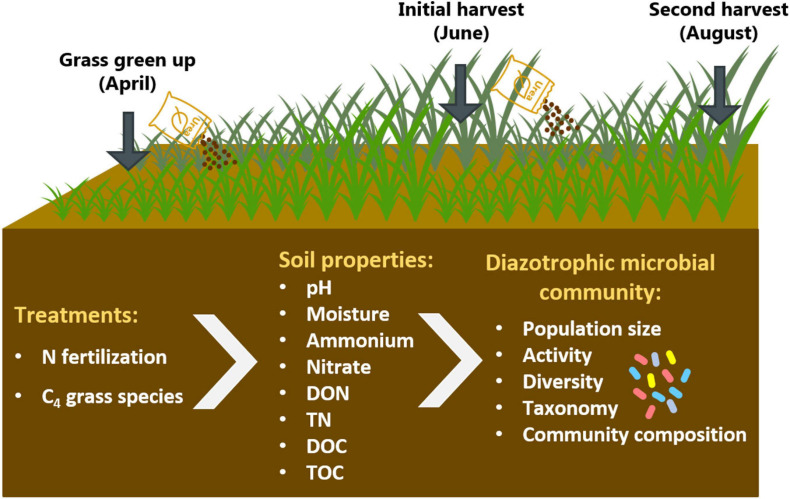
Schematic overview of the study.

In this study, we explored the impact of N fertilization on soil diazotrophic microbial communities under different native C_4_ grass systems in infertile, strongly leached Ultisols. There were two main objectives in our study: (i) determine the effects of N fertilization rate and C_4_ grass species on diazotrophic microbial communities; (ii) identify soil properties most related to diazotroph abundance, activity, diversity, and community composition. We hypothesized that: (1) increased N availability by N fertilization would suppress the abundance, activity, and diversity of diazotrophs, and excessive N fertilization would alter the community structure of diazotrophs; (2) because of the higher N use efficiency of big bluestem, the abundance, activity, and diversity of diazotrophs would be higher in big bluestem stands compared to stands dominated by switchgrass, and the diazotrophic microbial communities would be more responsive to N fertilization under switchgrass; (3) N fertilization would reduce the abundance, activity, and diversity of diazotrophs, and change diazotrophic microbial community due to increased soil N availability (ammonium and nitrate) and decreased soil pH. The study was conducted at a native grassland small plot experiment using two C_4_ grass species and three N fertilization rates. To examine the dynamics of soil diazotrophs, we used quantitative polymerase chain reaction (qPCR), quantitative reverse transcription PCR (qRT-PCR), and high-throughput amplicon sequencing to target N fixation gene *nifH* (Figure 1).

## Materials and Methods

### Study Site, Experimental Design, and Sample Collection

This study was conducted at an experimental field cultivated with native C_4_ grasses. This field was established in 2013 at the University of Tennessee East Tennessee AgResearch and Education Center (ETREC) in Knoxville, TN, United States (35.53° N, 83.06° W). Soils at this site are classified as sandy loam (fine-loamy, mixed, semiactive, thermic Typic Hapludult). The field was arranged in a randomized complete block design with split-plot treatment arrangements with two native C_4_ grass species (switchgrass [*Panicum virgatum*, SG] and big bluestem [*Andropogon gerardii*, BB]) being the main plot treatment and three N application rates (0, 67, and 202 kg N ha^–1^; hereafter referred to as 0N, 67N, and 202N) as the sub-plot treatment. These crossed treatments resulted in six cropping system combinations. Each treatment combination had three replicated plots. Sub-plots were 1.8 × 7.6 m in size. Grasses were planted in 2013, and N was applied as urea starting in 2014. Other fertilizers (phosphorus, potassium) and lime were applied in previous years at the recommended rates based on annual soil test results from the University of Tennessee Institute of Agriculture (UTIA) Soil, Plant and Pest Center (Nashville, TN, United States), but were not applied the year of this study. In addition, 2,4-dichlorophenoxyacetic acid (3.5 L ha^–1^) and Cimarron (52.5 g ha^–1^) herbicides were sprayed once per year for weed control. Plots were harvested in June and August using a Carter forage harvester (Carter Manufacturing Company, Inc., Brookston, IN, United States) with a 91.4-cm cutting width at a 20.3-cm cutting height.

Soil samples were collected from each sub-plot at three times during the growing season in 2019: grass green up (G, late April, 1 week before the first N fertilization), first grass harvest (H1, late June, within 1 week after harvest and before the second N fertilization), and second grass harvest (H2, Mid-August, within 1 week before the second harvest). Six soil subsamples (2.5 × 10 cm cores) were collected from each experimental sub-plot along the center-line of the long-axis to minimize influence from adjacent plots. Soil probes were sterilized with 70% ethanol after each sub-plot sampling. Soil subsamples from sub-plots were composited and stored in Ziplock^®^ bags and transported to laboratory on ice immediately after sampling. Then, composited samples were sieved through 2-mm mesh. A subsample of 10 g sieved soil per sub-plot was stored in −80°C freezer for DNA and RNA extraction, and the remainder of the soil sample was used for the analysis of soil physico-chemical properties.

### Soil Physico-Chemical Properties

Soil pH was measured in 1:2 soil:water suspension using pH electrode (Ultrabasic, Denver Instrument, Bohemia, NY, United States). Soil water content (SWC) was determined by weighing the fresh soil sample, drying it at 105°C for 48 h, reweighing, and calculating the weight of water lost as a percentage of the weight of the dried soil sample. Air-dried soil was pulverized and analyzed for total organic carbon (TC) and total nitrogen (TN) by dry combustion using an Elementar vario MAX cube (Elementar, Langenselbold, Germany). Dissolved organic C (DOC) and N (DON) were extracted in Milli-Q water and analyzed using an Elementar vario TOC cube in liquid mode. For soil NH_4_^+^-N and NO_3_^–^-N concentrations, 5 g fresh soil was extracted in 20 ml of 0.5 M K_2_SO_4_ solution by shaking on a reciprocating shaker at 150 rpm for 4 h. The solution was filtered through a Whatman GF/B filter (0.9 to 1.2 μm pore size) into clean container to collect the filtrate. The soil NH_4_^+^-N and NO_3_^–^-N concentration were analyzed by a Berthelot reaction-based spectrophotometric method (Rhine et al., 1998; Doane and Horwáth, 2003) using a microplate reader (Synergy HT, BioTek, Winooski, VT, United States).

### Soil Nucleic Acid Extraction and cDNA Synthesis

Soil genomic DNA was extracted from 0.25 g soil from each sample using the DNeasy PowerSoil Kit (Qiagen, Hilden, Germany) according to the manufacturer’s protocol. DNA was quantified using the NanoDrop One spectrophotometer (NanoDrop Technologies, Wilmington, DE, United States). Extracted DNA samples were stored at −20°C for qPCR amplification and sequencing.

Soil total RNA was extracted using a RNeasy PowerSoil Total RNA Kit (Qiagen, Hilden, Germany) according to the manufacturer’s protocol. RNA was eluted in RNase-free water and stored at −80°C. The quality was checked by 1% agarose gel electrophoresis and NanoDrop One spectrophotometry (NanoDrop Technologies, Wilmington, DE, United States). Samples were checked for DNA contamination by amplifying *nifH* genes using PCR and subsequent agarose gel electrophoresis. Only samples that did not have a PCR product (i.e., did not have DNA contamination) were used for reverse transcription. cDNA was produced from template RNA using SuperScript IV Reverse Transcriptase (Invitrogen, Paisley, United Kingdom) according to the manufacturer’s protocol. Random hexamer primers (Invitrogen) were used at a final concentration of 2.5 μM per reaction. cDNA samples were stored at −20°C for qRT-PCR amplification.

### Quantitative Analysis of *nifH* Genes and Gene Transcripts

Quantitative PCR (qPCR) of *nifH* genes and transcripts were performed on a CFX96 Optical Real-Time Detection System (Bio-Rad, Laboratories Inc., Hercules, CA, United States) using the primer pair IGK3 (5′-GCIWTHTAYGGIAARGGIGGIATHGGIAA-3′) and DVV (5′-ATIGCRAAICCICCRCAIACIACRTC-3′) (Gaby and Buckley, 2012). The 20-μl qPCR reaction mixture contained 10 μl Maxima SYBR Green qPCR Master Mix (2×) (Thermo Scientific, CA, United States), 1 μl of primer IGK3 (final concentration of 10 μM), 1 μl of primer DVV (final concentration of 10 μM), 2.5 μl DNA or cDNA template, and 5.5 μl PCR grade nuclease free water. The thermal conditions were as follows: initial denaturation at 95°C for 10 min, followed by 40 cycles of denaturation at 95°C for 30 s, annealing at 58°C for 1 min and extension at 72°C for 1 min, and final extension at 72°C for 10 min. Ten-fold serial dilutions (10^2^ to 10^8^ copies μl^–1^) of the plasmids containing *nifH* genes made through molecular cloning by the TOPO TA Cloning kit (Thermo Scientific, CA, United States) were used during qPCR assays to generate standard curves for quantifying *nifH* genes and transcripts in soil samples. Plasmids containing *nifH* genes amplified from environmental samples, described in our previous study (Hu et al., 2021), were used as standards. Following the reactions, a melt curve analysis was performed to confirm the specificity of the PCR product.

### *nifH* Gene Amplicon Sequencing

Soil DNA samples were diluted to 10 ng μl^–1^ for *nifH* gene amplification. Two-step PCR was used for *nifH* amplicon sequencing library preparation. For the first step, *nifH* genes were amplified from diluted DNA samples with primers IGK3/DVV containing Illumina-compatible adapters (5′-TCGTCGGCAGCGTCAGATGTGTATAAGAGACAG for IGK3 and 5′-GTCTCGTGGGCTCGGAGATGTGTATAAGAGACAG for DVV), following manufacturers’ recommendations (Klindworth et al., 2013). The amplifications were performed in a 25-μl mixture containing 12.5 μl DreamTaq Green PCR Master Mix (2×) (Thermo Scientific, CA, United States), 1 μl of each primer (final concentration of 0.4 μM), 2.5 μl of template DNA, and 8 μl of PCR grade nuclease-free water. The PCR thermal cycling conditions were 95°C for 3 min, followed by 35 cycles of 95°C for 30 s, 58°C for 1 min and 72°C for 1 min, followed by 72°C for 10 min. Agarose gel electrophoresis (1.5% gel) was applied to confirm the presence of a PCR product. Then, the PCR products were purified using SparQ PureMag Beads (Quantabio, MA, United States). For the second step, index PCR was performed in a 50-μL reaction which added unique indexes to each sample, containing 25 μl KAPA HiFi HotStart ReadyMix (2×) (Kapa Biosystems, MA, United States), 5 μl of each forward/reverse NexteraxT index/primer, 5 μl of PCR product from the first step as template DNA, and 10 μl of PCR grade nuclease-free water. The PCR amplification conditions were: denaturation at 95°C for 3 min, followed by eight cycles of 95°C for 30 s, 55°C for 30 s and 72°C for 30 s, and a final extension at 72°C for 5 min. SparQ PureMag Beads were used to purify the final indexed PCR products. The final PCR products were quantified by NanoDrop and pooled and diluted to 25 ng μl^–1^ to run on an Agilent Bioanalyzer to ensure quality for sequencing. Finally, 2 × 275-bp paired-ends sequencing was conducted at the University of Tennessee Genomics Core laboratory on an Illumina Miseq system (Illumina, CA, United States) with the Miseq reagent kit version 3 (1000 cycles) at 4 pM library loading concentration and with a 20% PhiX DNA (Illumina) included in the sequencing run.

### Bioinformatics Analysis

The generated raw *nifH* gene sequences were processed using Mothur v.1.39.5 (Schloss et al., 2009). Forward and reverse reads were merged using the make.contigs command. Primer sequences were trimmed using trim.seqs. Reads were removed if they contained <10 bp overlaps, ambiguous bases, homopolymers >7 bp, or were shorter than 300 bp using screen.seqs. Chimeras were removed by Chimera.uchime using a manually created database containing 7887 *nifH* gene reference sequences from Functional Gene Pipeline and Repository (FunGene^1^) (Fish et al., 2013). The remaining sequences were compared against a curated *nifH* gene database and its associated taxonomy annotation database (Gaby and Buckley, 2014; Gaby et al., 2018) using BLAST (Altschul et al., 1990) to identify cluster IV/V sequences; all cluster IV/V paralogs were removed by command get.seqs in Mothur. All libraries were subsampled to 5788 reads for downstream analyses. Operational taxonomic units (OTUs) of *nifH* were classified with 95% nucleotide sequence similarity cutoff using vsearch in QIIME2 (Bolyen et al., 2019). Singletons, OTUs appearing only once across all samples, were removed. The representative sequences of *nifH* OTUs were assigned to taxa using the *nifH* database of Gaby and Buckley (2014) and GenBank database. An OTU was assigned to either order (BLAST hit, <75%), family (<88.1%), genus (<91.9%), species (≥91.9%), or unclassified (no BLAST hit) (Gaby et al., 2018). The raw sequencing data can be accessed from NCBI Sequence Read Archive (SRA) Database, BioProject No. PRJNA687872.

### Statistical Analysis

In this study, the alpha- and beta-diversity of diazotrophs, *nifH* OTUs, and taxonomy data were derived from *nifH* amplicon sequencing data. The *nifH* OTU abundance refers to relative abundances in the sequence libraries. The *nifH* gene and transcript abundances were absolute abundances measured by qPCR and qRT-PCR, respectively. The alpha- and beta-diversity indices were calculated with QIIME2-2019.7 based on *nifH* amplicon OTU counts. Chao1 estimates of richness (Chao, 1984), observed number of *nifH* OTUs, Faith’s phylogenetic diversity (Faith, 1992), Shannon index, and Pielou’s evenness were calculated as metrics of alpha-diversity. Beta-diversity was quantified based on weighted-UniFrac distances (Lozupone et al., 2007). Permutational multivariate analysis of variance (PERMANOVA) based on the weighted-UniFrac distance matrix was used to test the differences among diazotrophic microbial community structures by treatment factors. A neighbor-joining phylogenetic tree was constructed by QIIME using representative sequences of 224 abundant *nifH* OTUs (OTUs with total reads ≥ 100), together with the relative abundance of each OTU in different treatment groups, and was visualized using Interactive Tree of Life (iTOL, v5) (Letunic and Bork, 2011).

Principal coordinates analysis (PCoA) based on weighted-UniFrac distances was performed in open-source software R (version 3.6.1) (R Core Team, 2013) with packages vegan (version 2.5-5) (Oksanen et al., 2020), phyloseq (version 1.28.0) (McMurdie and Holmes, 2013), and ggplot2 (version 3.3.1) (Wickham, 2011) to display the distribution of diazotrophic microbial community structures. Double principal coordinate analysis (DPCoA) (Pavoine et al., 2004) based on beta-diversity and taxonomy information of *nifH* OTUs was performed in R with packages vegan, phyloseq, and ggplot2 to visualize both distribution pattern of samples and *nifH* OTU optima. Redundancy Analysis (RDA) was conducted with vegan to model major factors (experimental treatments, soil physico-chemical properties, alpha-diversity of diazotrophs, and *nifH* gene and transcript abundances) shaping diazotrophic microbial community structures. Indicator species analysis based on the relative abundances of the top 224 abundant *nifH* OTUs was conducted with the multipatt function (number of permutations = 999) implemented in the R package indicspecies (version 1.7.9) (De Caceres et al., 2016) to identify taxon-habitat association patterns. This procedure returns an indicator value index that represent the strength of the association between a *nifH* OTU and a treatment or treatment combinations, with larger value indicating stronger association. The *nifH* OTUs significantly (*p*-value <0.05) associated with certain treatments or treatment combinations were identified as representative, or indicator *nifH* OTUs. Association network analysis, which reveals significant (*p* < 0.05) associations between *nifH* OTUs and the treatment(s) in which they were detected and/or abundant, was conducted using the interactive platform Gephi (WebAtlas, Paris, France) with Force Atlas placement algorithm.

A mixed model ANOVA within the GLIMMIX procedure in SAS 9.4 (SAS Institute, Cary, NC, United States) was performed to test the effects of sampling time, N fertilization rate, and grass species on soil properties, *nifH* gene and transcript abundances, and alpha-diversity of diazotrophic microbial communities. The abundances of *nifH* genes and transcripts were log transformed to achieve normal distributions. The fixed effects included sampling time, N fertilization rate, and grass species, as well as their interactions. Block was included as a random effect. A *post hoc* least significant difference (LSD) method was used to compare the means of groups.

Spearman correlation analysis was performed in IBM SPSS Statistics v26 to evaluate the correlation among *nifH* gene and transcript abundances, alpha-diversity of diazotrophic microbial communities, and soil physico-chemical parameters, as well as the correlations between *nifH* gene and transcript abundances and relative abundance of abundant *nifH* OTUs. Correlation coefficient *p* values were adjusted with a multiple comparison correction (BH method) in the stats package (version 4.0.3) within R.

Structural equation modeling (SEM) was performed with the IBM SPSS Amos 27.0 software to characterize the interaction among sampling time, grass species, N fertilization rate, soil physicochemical properties, *nifH* gene and transcript abundances, as well as alpha-diversity of diazotrophs and predominant diazotrophic taxa (orders). We followed the procedures of developing and modifying a structural equation model reported in Li et al. (2019). Briefly, we proposed a hypothesized model according to existing research and theory, tested if important pathways were left out and if the existing pathways were significant, and then revised the hypothesized model by adding missing pathways and dropping insignificant pathways in consideration of model fit and scientific rationality. Path coefficients were tested by maximum likelihood estimation at *p* ≤ 0.05. Multivariate normality was evaluated by Kurtosis value ≤ 7. Chi-square test of model fit, the Chi-square to degree of freedom (Df) ratio (Chi-square/Df), root mean square error of approximation (RMSEA), Tucker-Lewis index (TLI), incremental fit index (IFI), and comparative fit index (CFI) were used to determine model goodness-of-fit (Li and Schaeffer, 2020). A well fit model should have a Chi-square/Df value in the range of 1.0 to 3.0 with probability level >0.05, RMSEA value <0.08, and fit indices (TLI, IFI, and CFI) both >0.95 (Hooper et al., 2007; Blunch, 2012; Kline, 2015).

Raw data on soil properties and *nifH* gene and transcript abundances are available in the Supplementary Material.

## Results

### Soil Properties

Soil water content (SWC), dissolved organic C (DOC), and dissolved organic N (DON) showed significant differences by sampling time (*p* < 0.001 for SWC and DOC; *p* = 0.006 for DON) (Table 1 and Supplementary Table 1). The SWC was highest (0.302 ± 0.005 g H_2_O g^–1^ dry weight soil [gdw^–1^]) at initial grass harvest and lowest (0.150 ± 0.004 g H_2_O gdw^–1^) at second grass harvest whereas both DOC and DON were highest (223.00 ± 5.47 μg gdw^–1^ and 22.44 ± 1.98 μg gdw^–1^, respectively) after second grass harvest and lowest (163.85 ± 4.07 μg gdw^–1^ and 16.16 ± 0.65 μg gdw^–1^, respectively) after initial grass harvest (Supplementary Table 1).

**TABLE 1:** Results of mixed model ANOVA (based on GLIMMIX procedure in SAS) testing effects of agricultural season, nitrogen fertilization rate and grass species on soil properties and *nifH* gene and transcript abundances.

**Effect**	**pH**	**SWC (g H_2_O gdw^–1^)**	**NH_4_^+^-N (μ g gdw^–1^)**	**NO_3_^–^-N (μ g gdw^–1^)**	**DOC (μ g gdw^–1^)**	**DON (μ g gdw^–1^)**	**Total C (mg gdw^–1^)**	**Total N (mg gdw^–1^)**	**C:N ratio**	***nifH* gene (copies gdw^–1^)**	***nifH* transcript (copies gdw^–1^)**
Season	3.26	388.39***	23.06***	9.00***	57.99***	6.10**	0.43	1.11	0.98	57.42***	17.20***
Nitrogen	11.70***	0.31	0.01	31.14***	7.71**	1.20	0.88	2.44	2.15	0.43	2.31
Grass	11.53**	0.01	0.04	1.95	0.20	1.91	0.11	0.82	2.01	0.73	0.02
Nitrogen × grass	0.06	3.75*	1.09	0.17	2.62	1.06	1.13	0.08	4.35*	1.55	0.13
Season × nitrogen	0.59	0.89	3.40*	1.41	1.48	1.64	1.58	1.79	0.24	0.17	1.88
Season × grass	1.99	0.35	3.05	5.42**	0.98	0.83	1.22	1.52	0.24	0.41	1.15
Season × nitrogen × grass	0.13	0.57	1.35	1.61	0.21	1.13	0.84	0.53	0.26	0.44	1.22

*F*-values are reported. Significance level: *0.01 < *p*-value ≤ 0.05; **0.001 < *p*-value ≤ 0.01; ***p-value ≤ 0.001. SWC, soil water content; DOC, dissolved organic C; DON, dissolved organic N.

Several soil properties showed difference under different N fertilization rates and grass species (Table 1). Soil nitrate (NO_3_^–^-N) concentrations was lower under 0N (0.39 ± 0.16 μg gdw^–1^) and higher under 202N (2.77 ± 0.39 μg gdw^–1^). Soil pH was higher under 0N (6.36 ± 0.05) and lower under 202N (6.12 ± 0.05). DOC was higher under 0N (210.18 ± 8.60 μg gdw^–1^) and lower under 202N (186.07 ± 5.23 μg gdw^–1^). Soil pH was higher under switchgrass (6.32 ± 0.05) compared to big bluestem (6.19 ± 0.03) (*p* < 0.05). Soil water content was higher under N fertilization (67N, 202N) in switchgrass plots while it was lower under N fertilization in big bluestem plots. C:N ratio was not affected by N fertilization in big bluestem plots but was lower under 67N and 202N (11.63 ± 0.10 and 11.53 ± 0.09, respectively) compared to 0N (12.36 ± 0.36) in switchgrass plots (Supplementary Table 1).

The difference in NH_4_^+^-N concentrations among N fertilization rates depended on the sampling time (Table 1 and Supplementary Table 1): at grass green up, NH_4_^+^-N concentration was higher under 0N (12.29 ± 1.26 μg gdw^–1^) and lower under 202N (9.54 ± 0.38 μg gdw^–1^) whereas at both initial and second grass harvest, NH_4_^+^-N was lower under 0N (9.36 ± 1.14 and 6.46 ± 0.62 μg gdw^–1^, respectively) and higher under 202N (11.71 ± 0.84 and 7.61 ± 0.61 μg gdw^–1^, respectively). The interaction between sampling time and grass species was significant for NO_3_^–^-N concentration (Table 1 and Supplementary Table 1): under switchgrass, NO_3_^–^-N concentrations showed no difference among different sampling times, while under big bluestem, NO_3_^–^-N was higher at initial grass harvest (2.47 ± 0.78 μg gdw^–1^) compared to grass green up (1.12 ± 0.40 μg gdw^–1^) and second grass harvest (0.31 ± 0.15 μg gdw^–1^).

### Abundances of *nifH* Genes and Transcripts

**FIGURE 2:**
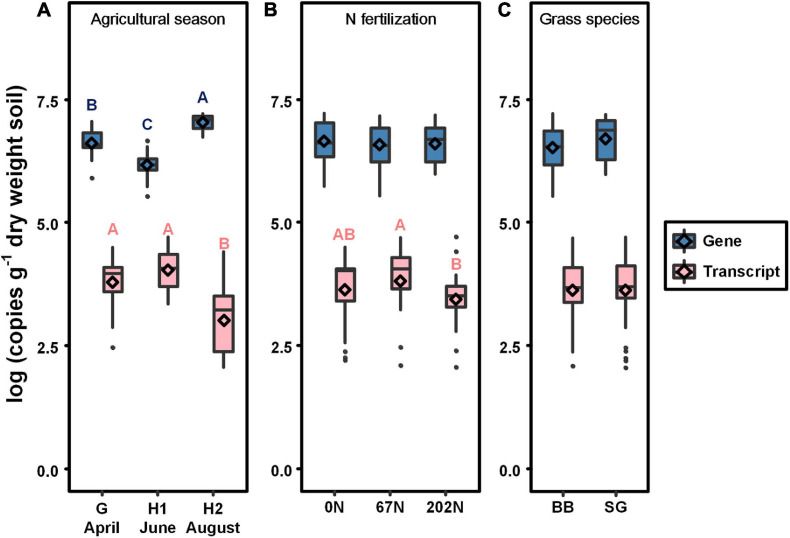
Absolute abundance of *nifH* gene (blue) and transcript (pink) in relation to agricultural seasons **(A)**, nitrogen fertilization rates **(B)**, and grass species **(C)**. Boxes represent the interquartile range (IQR). Lines within boxes represent median values. Whiskers represent the range between minimum and maximum values. Dots represent outliers outside of this range. Diamonds represent mean values. Different letters above boxes indicate significant differences between treatment levels within groups by comparing means using least significant difference (LSD) tests (α = 0.05). G, grass green up; H1, initial grass harvest; H2, second grass harvest; 0N, no N fertilization; 67N, 67 kg N ha^–1^ fertilization; 202N, 202 kg N ha^–1^ fertilization; BB, big bluestem; SG, switchgrass.

*nifH* gene and transcript abundances, representing population size and activity of diazotrophs, varied among sampling times (*p* < 0.0001 for both) but not among N fertilization rates or grass species; additionally, there were no significant interactions among sample time, N fertilization rate, and grass species (Table 1 and Figure 2). The mean abundance of *nifH* genes was lower after initial grass harvest (1.8 × 10^6^ copies gdw^–1^) and higher at second harvest (1.2 × 10^7^ copies gdw^–1^) (Figure 2A). The mean abundance of *nifH* gene transcripts was higher after initial harvest (1.6 × 10^4^ copies gdw^–1^) and lower at second grass harvest (3.5 × 10^3^ copies gdw^–1^) (Figure 2A). Compared to 67N, the abundance of *nifH* transcripts was lower under 202N (Figure 2B). No significant difference of gene or transcript abundance was observed between switchgrass and big bluestem plots (Figure 2C).

### Diazotrophic Microbial Structure and Community

**FIGURE 3:**
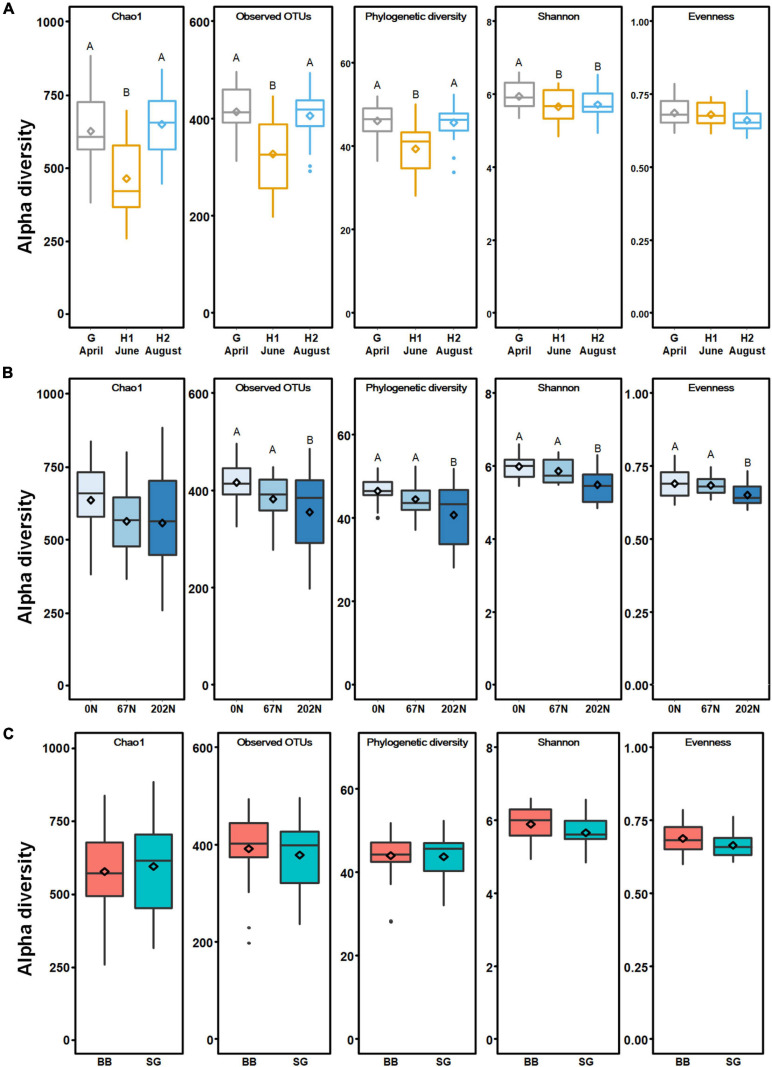
Alpha-diversity of diazotrophic microbial communities in relation to agricultural seasons **(A)**, nitrogen fertilization rates **(B)**, and grass species **(C)**. Boxes represent the interquartile range (IQR). Lines within boxes represent median values. Whiskers represent the range between minimum and maximum values. Dots represent outliers outside of this range. Diamonds represent mean values. Different letters above boxes indicate significant differences between treatment levels within groups by comparing means using least significant difference (LSD) tests (α = 0.05). The letters were only shown in groups that have significant effects. G, grass green up; H1, initial grass harvest; H2, second grass harvest; 0N, no N fertilization; 67N, 67 kg N ha^–1^ fertilization; 202N, 202 kg N ha^–1^ fertilization; BB, big bluestem; SG, switchgrass.

A total of 2595 *nifH* OTUs were generated at 95% sequence similarity after subsampling to 5788 reads per sample. N fertilization rate significantly affected diazotrophic alpha-diversity (Supplementary Table 2). The richness indices (Chao1, observed OTUs, and phylogenetic diversity) were similarly high at grass green up (G) and second grass harvest (H2) but were lower at initial grass harvest (H1) (*p* < 0.05) (Figure 3A). The Shannon index reflecting both richness and evenness was similarly low at the initial and second grass harvest and was higher at grass green up (*p* < 0.05) (Figure 3A). Alpha-diversity indices were lower under N fertilization than those under no N fertilization (Figure 3B). The mean values of observed OTUs, phylogenetic diversity, Shannon index, and Pielou’s evenness under 202N were lower than that under 0N (*p* < 0.05) (Figure 3B). Grass species did not affect diazotrophic alpha-diversity (Figure 3C). Moreover, no interaction effect of sample time, N fertilization, and grass species on the alpha-diversity of diazotrophic microbial community was observed (Supplementary Table 2).

**FIGURE 4:**
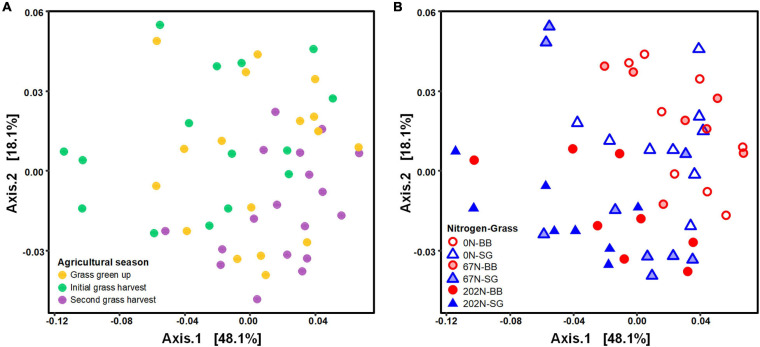
Principal coordinates analysis (PCoA) of weighted-UniFrac distances between diazotrophic microbial communities colored by agricultural season **(A)** and the combination of N rate and grass species **(B)**. 0N, no N fertilization; 67N, 67 kg N ha^–1^ fertilization; 202N, 202 kg N ha^–1^ fertilization; SG, switchgrass; BB, big bluestem.

Nitrogen fertilization rate and grass species had significant effects on the structure of diazotrophic microbial community (Table 2). N fertilization rate had a stronger effect (PERMANOVA *R*^2^ = 0.210, *p* = 0.001) than sampling time (*R*^2^ = 0.148 *p* = 0.001) and grass species (*R*^2^ = 0.066, *p* = 0.005) on diazotrophic microbial community structure (Table 2 and Figure 4). Results of pairwise PERMANOVA showed that the diazotrophic microbial community structure at the two grass harvest times were significantly different from each other (*q*-value ≤ 0.05), but both showed no significant difference from grass green up (Figure 4A and Supplementary Table 3). Diazotrophic community composition in 0N and 67N treatments differed from 202N (Figure 4B and Supplementary Table 3). In addition, the response of diazotrophic microbial community composition to N-rate differed between the two grass species: under big bluestem, communities with 67N (67N-BB) differed from 202N (202N-BB); under switchgrass, the communities with 67N (67N-SG) showed no significant difference from 202N (202N-SG) (Figure 4B and Supplementary Table 3).

**TABLE 2:** Results of PERMANOVA examining the effects of season, N fertilization rate, and grass species on diazotrophic community composition, based on weighted-UniFrac distances of *nifH* OTUs.

**Factor**	**Df**	**F.Model**	***R*^2^**	***p*-value^†^**
Season	2	5.413	0.148	**0.001**
Nitrogen	2	7.697	0.210	**0.001**
Grass	1	4.804	0.066	**0.005**
Season × nitrogen	4	1.081	0.059	0.352
Season × grass	2	0.445	0.012	0.920
Nitrogen × grass	2	1.667	0.046	0.110
Season × nitrogen × grass	4	0.426	0.023	0.982
Residuals	32		0.437	

*^†^Bold values indicate *p*-value ≤ 0.05.*

### Phylogenetic Affiliations of Diazotrophic Communities

The phylum Proteobacteria had the highest relative abundance in the diazotrophic communities, with 1809 *nifH* OTUs and 95.1% of the total reads belonging to Proteobacteria (Supplementary Figure 1). Cyanobacteria and Firmicutes accounted for 1.0% and 0.7% of the total reads, respectively (Supplementary Figure 1). The *nifH* OTU with highest relative abundance (7.6% of total reads) was classified to family Sphingomonadaceae (class α-Proteobacteria). The next three most abundant *nifH* OTUs all classified as family Bradyrhizobiaceae (order Rhizobiales, class α-Proteobacteria), and accounted for 7.4%, 6.4%, and 6.2% of total reads, respectively.

**FIGURE 5:**
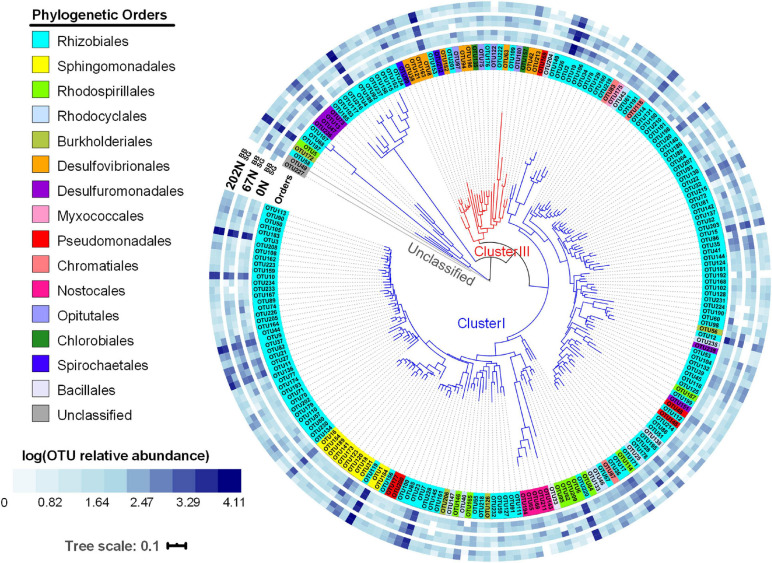
Circular neighbor-joining phylogenetic tree of the top 224 abundant *nifH* OTUs (OTUs with ≥ 100 reads). Tree leaves on the inner circle highlighted by colors show the affiliation to different phylogenetic orders. The outer blue-and-white heatmap circles show the logarithmic value of mean OTU relative abundances in percentages in different N fertilization rates and grass species (all three seasons combined). 0N, no N fertilization; 67N, 67 kg N ha^–1^ fertilization; 202N, 202 kg N ha^–1^ fertilization; SG, switchgrass; BB, big bluestem.

A phylogenetic tree based on a neighbor-joining method for the 224 most abundant *nifH* OTUs (OTUs with ≥ 100 reads) showed that 149 *nifH* OTUs belong to order Rhizobiales (Figure 5). In our study, the abundant *nifH* OTUs belonged to cluster I or cluster III: 198 of the abundant *nifH* OTUs belonged to phylum Proteobacteria, Cyanobacteria, or Firmicutes and affiliated with cluster I; 24 of the abundant *nifH* OTUs belonged to phylum Proteobacteria, Verrucomicrobia, Spirochaetes, or Chlorobi and affiliated with cluster III (Figure 5). The relative abundances of *nifH* OTUs in cluster I remained unchanged with N fertilization rate (5088 ± 66, 5002 ± 65, and 4961 ± 59 sequences under 0N, 67N, and 202N, respectively), while cluster III OTUs increased in relative abundance with N fertilization rate (266 ± 45, 383 ± 65, and 557 ± 77 sequences under 0N, 67N, and 202N, respectively) (Figure 5).

### Indicator Species Under N Fertilization and Grass Species

**FIGURE 6:**
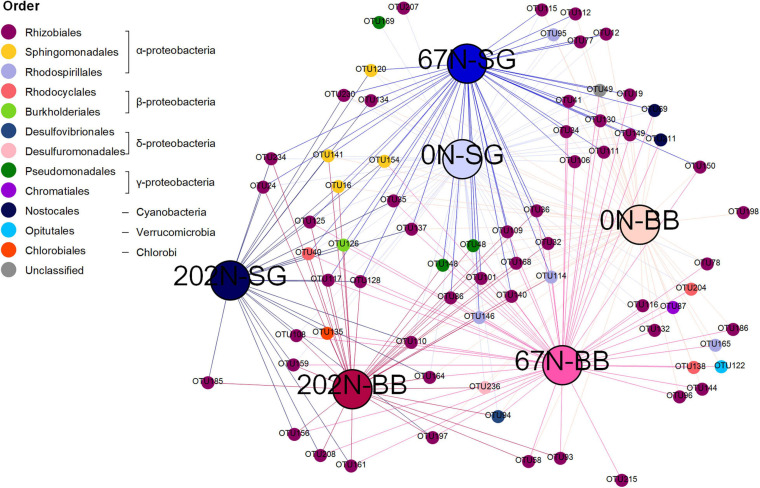
Association network showing significant (*p* < 0.05) positive associations between *nifH* OTUs and specific habitat under two different treatments [specific N fertilization rate (0N, no N fertilization; 67N, 67 kg N ha^–1^ fertilization; 202N, 202 kg N ha^–1^ fertilization) and grass species (SG, switchgrass; BB, big bluestem)]. Large nodes represent treatments. Small nodes represent *nifH* OTUs. Line colors reflect treatments. *nifH* OTUs were grouped to order level.

We identified 71 *nifH* OTUs significantly associated to one or more treatment combinations of N fertilization rate and grass species (Supplementary Table 4), which were represented in an association network (Figure 6). Many *nifH* OTUs were representative of certain N fertilization and/or grass species treatments. For example, one *nifH* OTU (OTU 185, belonging to Rhizobiales) was only detected under 202N (specificity value A = 1.000, fidelity value B = 0.588 *p* = 0.003) whereas OTU 69 (belonging to Nostocales) was only detected under 0N and 67N (A = 1.000, B = 0.697, *p* = 0.002). Moreover, an unclassified diazotroph (OTU 49) appeared in all plots under 0N and 67N (B = 1.000) and was largely (but not completely) restricted to these plots (A = 0.972). Two *nifH* OTUs (OTU 58 and OTU 93, both belonging to Rhizobiales) were significantly associated with big bluestem (BB) (*p* = 0.002 and 0.006, respectively). Some OTUs were significantly associated to only a single treatment combination (e.g., OTU 169 and OTU 207 for 0N combined with switchgrass (0N-SG); OTU 198 for 0N-BB; OTU 215 for 67N-BB).

**FIGURE 7:**
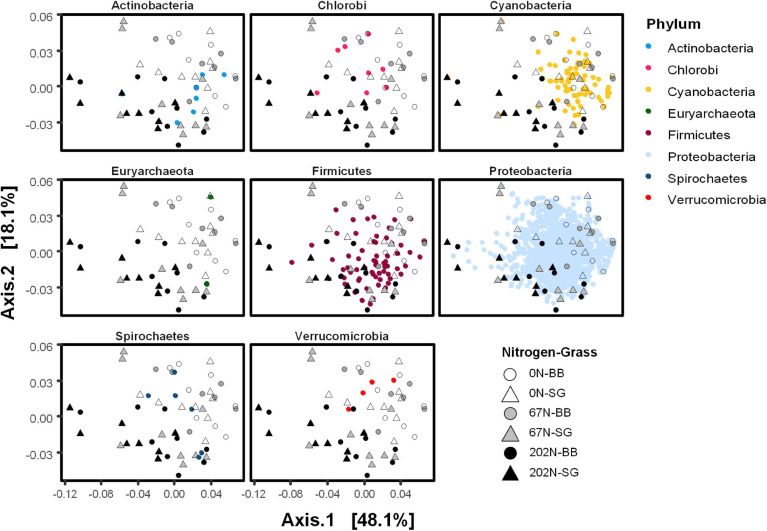
Double principal coordinate analysis (DPCoA) of weighted-UniFrac distance between diazotrophic microbial communities. Both samples and OTU optima were displayed. Small points on the ordination plots represent *nifH* OTUs and are colored by phylum. Large circle and triangle points on the ordination plots represent samples and are shaped by N fertilization rate and grass species. 0N, no N fertilization; 67N, 67 kg N ha^–1^ fertilization; 202N, 202 kg N ha^–1^ fertilization; SG, switchgrass; BB, big bluestem.

A double principal coordinate analysis (DPCoA) (Pavoine et al., 2004) showing both distribution pattern of samples and *nifH* OTU optima indicated that OTUs affiliated to Cyanobacteria were mainly found under 0N and 67N treatments rather than 202N, whereas OTUs affiliated to Firmicutes were mainly found under 67N and 202N (Figure 7). The OTUs affiliated to other phyla did not show a pattern by N fertilization rates. In addition, we observed that the relative abundance of δ-Proteobacteria was higher under 202N than under 0N and 67N regardless of sampling time and grass species, while γ-Proteobacteria was higher under 0N and 67N than under 202N (Supplementary Figure 1). The phylum Verrucomicrobia had higher relative abundance in big bluestem plots than in switchgrass plots (Supplementary Figure 1).

### Relationships Among Soil Properties, *nifH* Gene and Transcript Abundances, and Diazotrophic Microbial Structure and Community

**FIGURE 8:**
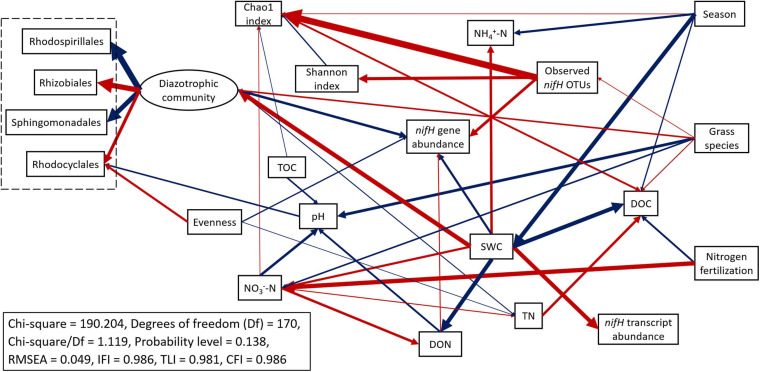
Structural equation modeling (SEM) of diazotrophic microbial abundance, functional activity, diversity, and predominant taxa (order level) with key soil parameters. Boxes represent observable variables. An ellipse represents the latent variable of community composition, which was inferred from the measured relative abundances of taxa in the community (dashed box). The four orders shown were selected because they had significant associations with the other factors in the model. Season refers to sampling time (grass green up, first harvest or second harvest). Red arrows indicate positive relationships. Blue arrows indicate negative relationships. Single headed arrows represent causal relationship (*p*-value < 0.05). The direction of arrow indicates the direction of causation. The width of arrow indicates the extent of effects, i.e., the standardized path coefficients proportional to the thickness of arrows. See Supplementary Table 6 for specific standardized path coefficients and Supplementary Table 7 for standardized total effects. SWC, soil water content; DOC, dissolved organic C; DON, dissolved organic N; TOC, total organic C; TN, total N.

The abundances of *nifH* genes and transcripts were negatively correlated (*R* = −0.349, *p* < 0.05). The abundance of *nifH* genes was negatively correlated to SWC (*R* = −0.781, *p* < 0.01) and soil inorganic N (NH_4_^+^-N: *R* = −0.510, *p* < 0.01; NO_3_^–^-N: *R* = −0.387, *p* < 0.01) but positively correlated to DOC (*R* = 0.728, *p* < 0.01) and DON (*R* = 0.510, *p* < 0.01). In contrast, the abundance of *nifH* gene transcripts was positively correlated to pH (*R* = 0.423, *p* < 0.01), SWC (*R* = 0.590, *p* < 0.01), and NH_4_^+^-N (*R* = 0.320, *p* < 0.05) but negatively correlated to DOC (*R* = −0.280, *p* < 0.05) (Supplementary Table 5). Structural equation modeling (SEM) was conducted to identify the impacts of treatments and soil parameters on the abundance, functional activity, diversity, and predominant community composition of diazotrophs (Figure 8 and Supplementary Table 6). SWC had a direct and strong positive effect on diazotroph activity (*nifH* transcript abundance) (standardized path coefficient = 0.602, *p*-value < 0.001) but had a direct negative effect on diazotroph abundance (reflecting by *nifH* gene abundance) (standardized path coefficient = −0.334, *p*-value < 0.001). SWC had a direct and strong positive effect on the diazotrophic community (standardized path coefficient = 0.677, *p*-value < 0.001). N fertilization rate directly and positively affected soil nitrate concentration (standardized path coefficient = 0.689, *p*-value < 0.001). The increased soil nitrate by N fertilization negatively affected soil pH (standardized path coefficient = −0.369, *p*-value = 0.001), which negatively affected the relative abundance of Rhodocyclales, one of the most abundant orders within diazotrophic communities (standardized path coefficient = −0.248, *p*-value = 0.011). Moreover, diazotroph abundances had positive association with observed richness (observed *nifH* OTUs) (standardized path coefficient = 0.427, *p*-value < 0.001). The standardized total effects obtained from structural equation modeling were shown in Supplementary Table 7. Compared to switchgrass, big bluestem increased the richness of diazotrophs and the relative abundance of Rhizobiales and Rhodocyclales but decreased both diazotrophs abundance and activity as well as the relative abundance of Sphingomonadales and Rhodospirillales. DON had a positive total effect on diazotroph abundance.

**FIGURE 9:**
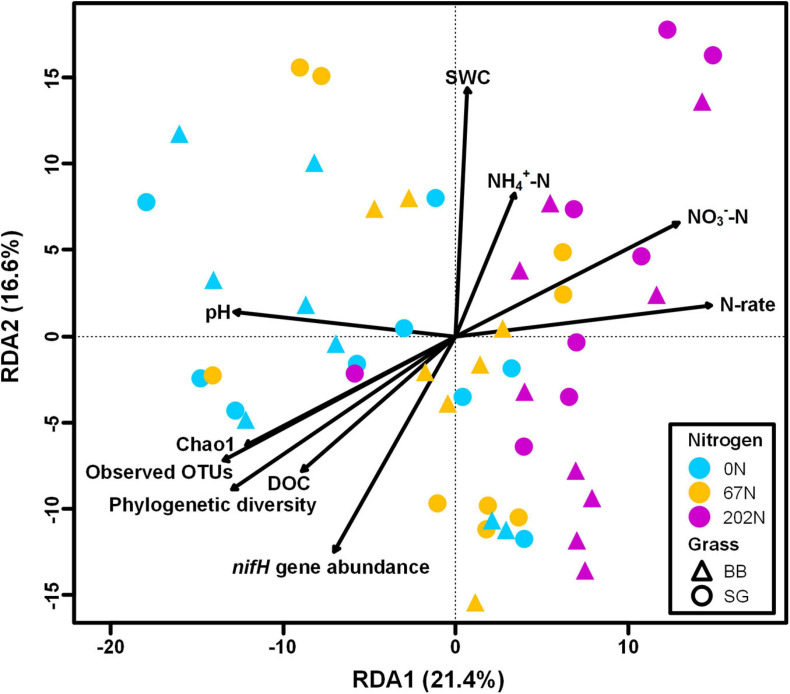
Redundancy analysis (RDA) of diazotrophic microbial community (circle and triangle symbols) with environmental factors, *nifH* gene and transcript abundances, and community diversity metrics as predictor variables; significant predictors are indicated with arrows. 0N, no N fertilization; 67N, 67 kg N ha^–1^ fertilization; 202N, 202 kg N ha^–1^ fertilization; SG, switchgrass; BB, big bluestem; SWC, soil water content; DOC, dissolved organic C; N-rate, N fertilization rate.

A redundancy analysis (RDA) showed that soil parameters, *nifH* gene and transcript abundances, agricultural season, N fertilization rate, grass species, and five alpha-diversity indices measured in this study explained a total of 68.4% of the variability of diazotrophic community composition, with the first two axes explaining 38.0% of this variability (Figure 9). The RDA showed that soil pH, SWC, NH_4_^+^-N concentration, NO_3_^–^-N concentration, DOC, *nifH* gene abundance, N fertilization rate, and richness indices of alpha-diversity significantly correlated to the variability of diazotrophic community (*p* < 0.05) (Figure 9).

Spearman correlation analysis showed that there were positive correlations between the richness indices (Chao1, observed OTUs, and Faith’s phylogenetic diversity) of diazotrophic bacteria and *nifH* gene abundance but no correlation between diazotrophic alpha-diversity and *nifH* transcript abundance (Supplementary Table 8). These positive correlations were stronger under switchgrass than under big bluestem (Supplementary Table 8). In addition, only the relative abundances of four *nifH* OTUs (OTU1, OTU5, OTU6, and OTU18) in the top 20 most abundant *nifH* OTUs were positively correlated to *nifH* gene abundance quantified by qPCR (Supplementary Table 9). These four *nifH* OTUs belonged to Sphingomonadales (OTU1 and OTU18) and Rhodospirillales (OTU5 and OTU6), indicating the variation of *nifH* gene abundances measured by qPCR may potentially depend on the variation of these two orders.

## Discussion

The primary objective of this study was to investigate the impacts of N fertilization rate and native grass species on the seasonal population size, activity, and diversity of diazotrophic microbial communities in a native C_4_ grass system dominated by infertile, strongly leached Ultisols using qPCR, qRT-PCR, and *nifH* gene amplicon sequencing. The population size, activity, and community composition of diazotrophs varied throughout the season, most likely due to variation of N and C availability (Pereira et al., 2013; Wang et al., 2016), soil temperature (Deslippe et al., 2005), precipitation (Yeager et al., 2012), and other environmental factors. The greatest abundance of diazotrophs, as indicated by *nifH* gene abundance, but lowest activity, as indicated by *nifH* transcript abundance, were observed at the second grass harvest in August, which we attributed to the lower SWC in summer months. In general, SWC is assumed to promote the activity of soil diazotrophs (Ritchie and Raina, 2016; Feng et al., 2019) as higher SWC decreases soil oxygen content (Brusseau et al., 2006) that inhibits nitrogenase activity (Dobereiner et al., 1972; Eady and Postgate, 1974). Here we observed that diazotroph abundances did not decrease in drier soils, perhaps because the most abundant diazotrophs detected in this study were classified as aerobes or facultative anaerobes (e.g., *Sphingomonas*, *Bradyrhizobium*, and *Rhodospirillum*) which would tolerate oxygenated soils. We observed a positive effect of SWC on diazotroph activity (*nifH* transcript abundance) in our structural equation modeling and correlation analysis. This is likely due to the inhibitory effect of oxygen on nitrogenase activity (Eady and Postgate, 1974).

Availability of C is another key factor that may affect the abundance of diazotrophs. We observed a positive correlation between DOC and *nifH* gene copies. Other studies have shown that decreased DOC may limit the growth of heterotrophic diazotrophs (Yin et al., 2013; Feng et al., 2019). One explanation for the lower DOC observed at first grass harvest (June) is that we sampled soils after mowing at first grass harvest but before mowing at second grass harvest. Mowing can decrease soil DOC due to (1) decreased C substrate supplied from photosynthesis and aboveground litter (Wan and Luo, 2003; Gilmullina et al., 2020); (2) increased plant root uptake of easily available C; and (3) decreased plant C flow to soil in the form of root exudates (Bardgett et al., 1999).

Nitrogen fertilization, especially at the highest N rate, resulted in higher soil NH_4_^+^ and NO_3_^–^ concentrations and lower soil pH and DOC. The lower DOC content at high N rate might be due to the promotion of N immobilization by soil microbes. In contrast to our hypothesis, no effect of N fertilization on the abundance of diazotrophs was observed, which could be explained several ways: (1) diazotrophs were able to get N from soil for growth and reproduction and did not need to rely on atmospheric N_2_ fixation; (2) the decreased soil pH by urea fertilization was not enough to suppress the abundance of diazotrophs; and/or (3) the abundant diazotrophs in this system were more nitrate-tolerant. Indeed, some species within the genus *Bradyrhizobium* are denitrifiers and can tolerate higher levels of nitrate (Gao et al., 2020). For instance, OTU 3 detected in our study is an abundant *nifH* OTU affiliated to the legume-root nodulating bacteria *Bradyrhizobium japonicum*. *B. japonicum* has capacity for complete denitrification and nitrate respiration (Bedmar et al., 2005; Vessey and Luit, 2011). High N fertilization rates (202N) may not have affected abundance of diazotrophs, but it did decrease their activity. This is likely because diazotrophs preferentially use easily available N (e.g., nitrate and ammonium) as this takes less energy to assimilate compared to fixing dinitrogen gas (Chapin et al., 2002; Smercina et al., 2019). Another possible reason for the decreased activity of diazotrophs may be the lower pH caused by high N fertilization rate. Diazotrophs typically have a pH optima of 7.0 to 7.5, and reduced activity when pH is lower than 6.7 (Roper and Smith, 1991; Limmer and Drake, 1996; Roper and Gupta, 2016; Han et al., 2019).

Nitrogen fertilization rate had a strong impact on diazotrophic microbial community composition, with significant changes in diazotrophic microbial community composition, species richness and evenness under high N fertilization. The high N fertilization rate shifted the community composition toward species better adapted to higher nitrate and lower soil pH. We observed that the *nifH* OTUs belonging to Firmicutes were mainly found under high N, where we also observed the lowest pH. The role of pH in shaping diazotrophic community has been observed in other ecosystems, such as alpine meadows (Wang Y. et al., 2017) and wheat fields (Fan et al., 2018). The relative abundance of cluster III *nifH* OTUs, which mainly affiliated with known obligate anaerobes, increased with N-rate. One possible explanation is that the cluster III diazotrophs may better tolerate high nitrate and/or low pH conditions. Specifically, OTU 8, OTU 38, and OTU 42 within cluster III belonged to Desulfovibrionales and increased in relative abundance with N-rate. This order contains sulfate-reducing bacteria that have been reported to reduce nitrate for growth in the absence of sulfate (Marietou et al., 2009; Sánchez-Andrea et al., 2015; Marietou and Boden, 2016).

According to our RDA analysis and SEM model, N fertilization influenced the variability of diazotrophic microbial community composition by increasing ammonium and/or nitrate concentration and decreasing soil pH. Unmeasured environmental factors, such as climatic factors (Han et al., 2019), and/or other soil nutrients such as phosphorus and potassium (Wang C. et al., 2017, Wang et al., 2020; Zou et al., 2020), may also contribute to the variation of diazotrophic community composition. Many studies have suggested that the metabolic functional potential of a community may be more sensitive to environmental factors than its composition (Raes et al., 2011; Louca et al., 2016; Nelson et al., 2016). We observed this in our SEM, which showed that the soil N concentrations, such as DON and nitrate, elevated by N fertilization, as well as SWC, directly and positively affected the population size of diazotrophs. The positive correlation of the diversity with abundance, but not activity, might be due to functional redundancy in these communities. Functional redundancy suggests that in a more diverse community, functionally similar bacteria may be active at different times, depending on conditions (Louca et al., 2016, 2018; Nelson et al., 2016).

## Conclusion

We documented the seasonal dynamics of diazotrophic abundance, activity, diversity, and community composition under two C_4_ grass species and three N fertilization rates. Our results showed that cluster I and III diazotrophs were dominant in the studied native C_4_ grass system. The population size and activity of diazotrophs were related to SWC and C availability over a growing season. Nitrogen fertilization rate had a stronger influence on diazotrophic community composition than grass species. Consistent with our hypotheses, compared to moderate N fertilization, excessive N fertilizer application decreased both alpha-diversity and activity of diazotrophic community, and altered diazotrophic community composition. This suggests excessive fertilization in native C_4_ grass systems may have negative consequences for biological N fixation and soil health. Moreover, soil pH, soil moisture, and C and N availability were related to the variability of diazotrophic community composition. Overall, our work revealed insights into important relationships between diazotrophic community abundance and diversity in the field, adding to our understanding of the response of soil diazotrophs to season, nitrogen fertilization, and grass species in native C_4_ grass systems.

## Data Availability Statement

The raw sequencing datasets for this study can be accessed from NCBI Sequence Read Archive (SRA) Database: https://cata log.data.gov/dataset/sequence-read-archive-sra, BioProject No. PRJNA687872. Raw data on soil properties and *nifH* gene and transcript abundances are available in the Supplementary Material.

## Author Contributions

JR, PK, SJ, and JD conceived and designed the study. JH, JR, and FY conducted the experiment. JH performed qPCR, qRT-PCR and amplicon sequencing work. JH, LL, and JD analyzed the data. JR, PK, and SJ supervised the project and provided guidance on the framing of questions and interpretation of findings. JH prepared the manuscript with all authors contributing to the drafts.

## Conflict of Interest

The authors declare that the research was conducted in the absence of any commercial or financial relationships that could be construed as a potential conflict of interest.

## Footnotes

1 http://fungene.cme.msu.edu/
