# Supplementary material for: Nitrogen Fertilization and Native C4 Grass Species Alter Abundance, Activity, and Diversity of Soil Diazotrophic Communities
Source: Front Microbiol. 2021 Jul 8;12:675693. doi: 10.3389/fmicb.2021.675693 (PMC8297707; doi:10.3389/fmicb.2021.675693)
Supplement: Supplementary file 1 [file Data_Sheet_1.docx]

***Supplementary Material***

# Supplementary Figures and Tables

## Supplementary Tables

**Table S1.** Soil properties in relation to agricultural season, N fertilization rate, and grass species. Different lowercase letters indicate significant differences between treatment levels within groups. The letters are only shown in groups that had significant differences based on mixed model ANOVA (GLIMMIX procedure in SAS). Means were compared using Least Significant Difference (LSD) tests (α = 0.05).

| Factor^†^ | pH | SWC^‡^  (g H_2_O g^-1^ dry soil) | NH_4_^+^-N  (μg g^-1^ dry soil) | NO_3_^-^-N  (μg g^-1^ dry soil) | Dissolved Organic C  (μg g^-1^ dry soil) | Dissolved Organic N  (μg g^-1^ dry soil) | Total C  (mg g^-1^ dry soil) | Total N  (mg g^-1^ dry soil) | C: N ratio |
| --- | --- | --- | --- | --- | --- | --- | --- | --- | --- |
| G (April) | 6.27±0.05 | 0.24±0.00^b*^ | 10.82±0.49^a^ | 1.51±0.30^a^ | 203.55±4.20^b^ | 18.67±0.56^ab^ | 1.82±0.03 | 21.74±0.43 | 11.94±0.11 |
| H1 (June) | 6.30±0.06 | 0.30±0.00^a^ | 10.57±0.60^a^ | 2.14±0.46^a^ | 163.85±4.07^c^ | 16.16±0.65^b^ | 1.76±0.06 | 21.18±0.68 | 12.05±0.15 |
| H2 (August) | 6.18±0.03 | 0.15±0.00^c^ | 7.16±0.35^b^ | 0.83±0.25^b^ | 223.00±5.47^a^ | 22.44±1.98^a^ | 1.84±0.05 | 21.66±0.51 | 11.81±0.12 |
| 0N | 6.36±0.05^a^ | 0.23±0.02 | 9.37±0.85 | 0.39±0.16^c^ | 210.18±8.60^a^ | 17.65±1.10 | 1.71±0.04 | 20.73±0.53 | 12.11±0.16 |
| 67N | 6.29±0.04^a^ | 0.23±0.02 | 9.53±0.57 | 1.13±0.19^b^ | 196.38±8.02^b^ | 19.07±0.95 | 1.82±0.04 | 21.74±0.49 | 11.94±0.12 |
| 202N | 6.12±0.05^b^ | 0.23±0.02 | 9.62±0.53 | 2.77±0.39^a^ | 186.07±5.23^b^ | 20.29±1.83 | 1.87±0.05 | 21.97±0.58 | 11.78±0.09 |
| SG | 6.32±0.05^a^ | 0.23±0.01 | 9.52±0.49 | 1.71±0.24 | 195.32±6.71 | 20.28±1.49 | 1.83±0.04 | 21.60±0.41 | 11.79±0.12 |
| BB | 6.19±0.03^b^ | 0.23±0.01 | 9.51±0.54 | 1.30±0.33 | 198.11±5.77 | 18.03±0.68 | 1.78±0.04 | 21.46±0.47 | 12.06±0.08 |
| 0N-SG | 6.48±0.09 | 0.22±0.03^b^ | 10.03±1.75 | 0.48±0.17 | 201.67±13.80 | 17.34±1.71 | 1.74±0.06 | 21.49±0.81 | 12.36±0.36^a^ |
| 67N-SG | 6.35±0.05 | 0.24±0.02^ab^ | 9.38±0.51 | 1.39±0.16 | 194.43±13.99 | 19.97±1.52 | 1.83±0.05 | 21.37±0.54 | 11.67±0.10^b^ |
| 202N-SG | 6.19±0.08 | 0.24±0.02^ab^ | 9.32±0.54 | 2.86±0.32 | 191.98±8.07 | 22.55±3.48 | 1.90±0.07 | 21.90±0.84 | 11.53±0.09^b^ |
| 0N-BB | 6.27±0.03 | 0.24±0.02^a^ | 8.93±0.87 | 0.34±0.25 | 215.85±11.24 | 17.86±1.51 | 1.70±0.06 | 20.22±0.69 | 11.94±0.12^ab^ |
| 67N-BB | 6.23±0.06 | 0.23±0.03^ab^ | 9.69±1.05 | 0.88±0.33 | 198.33±8.76 | 18.18±1.14 | 1.81±0.07 | 22.12±0.82 | 12.21±0.18^ab^ |
| 202N-BB | 6.06±0.04 | 0.22±0.02^b^ | 9.92±0.95 | 2.68±0.73 | 180.16±6.50 | 18.04±0.93 | 1.83±0.07 | 22.04±0.85 | 12.02±0.11^ab^ |
| G-0N | 6.35±0.10 | 0.25±0.01 | 12.29±1.26^a^ | 0.45±0.17 | 220.18±3.73 | 19.97±0.88 | 1.79±0.04 | 21.50±0.48 | 12.01±0.23 |
| G-67N | 6.31±0.05 | 0.24±0.00 | 10.87±0.51^ab^ | 1.20±0.21 | 204.94±6.82 | 18.24±0.69 | 1.77±0.04 | 21.10±0.71 | 11.95±0.20 |
| G-202N | 6.16±0.07 | 0.24±0.01 | 9.54±0.38^bc^ | 2.70±0.52 | 188.30±3.30 | 18.00±1.21 | 1.90±0.07 | 22.58±0.89 | 11.87±0.18 |
| H1-0N | 6.43±0.09 | 0.30±0.01 | 9.36±1.14^bcd^ | 0.67±0.44 | 169.15±6.71 | 15.21±1.12 | 1.60±0.06 | 19.68±1.00 | 12.31±0.43 |
| H1-67N | 6.38±0.09 | 0.31±0.01 | 10.43±1.10^ab^ | 1.59±0.39 | 159.77±9.47 | 16.23±1.30 | 1.75±0.07 | 21.16±1.06 | 12.05±0.15 |
| H1-202N | 6.12±0.12 | 0.30±0.01 | 11.71±0.84^a^ | 3.90±0.76 | 163.52±4.73 | 16.88±1.04 | 1.90±0.11 | 22.43±1.30 | 11.82±0.16 |
| H2-0N | 6.30±0.04 | 0.15±0.01 | 6.46±0.62^e^ | 0.06±0.04 | 241.21±5.54 | 17.78±2.79 | 1.75±0.10 | 21.01±1.16 | 12.00±0.18 |
| H2-67N | 6.19±0.06 | 0.15±0.01 | 7.30±0.57^de^ | 0.61±0.27 | 224.43±8.92 | 22.76±1.58 | 1.95±0.08 | 22.96±0.57 | 11.83±0.28 |
| H2-202N | 6.08±0.03 | 0.15±0.01 | 7.61±0.61^cde^ | 1.71±0.47 | 206.38±7.76 | 26.01±4.66 | 1.80±0.06 | 20.89±0.77 | 11.64±0.14 |
| G-SG | 6.34±0.08 | 0.25±0.00 | 11.30±0.90 | 1.95±0.42^ab^ | 202.78±4.65 | 19.57±0.72 | 1.89±0.05 | 22.14±0.64 | 11.75±0.15 |
| G-BB | 6.20±0.04 | 0.24±0.00 | 10.39±0.46 | 1.12±0.40^b^ | 204.24±7.05 | 17.86±0.79 | 1.76±0.03 | 21.38±0.60 | 12.11±0.15 |
| H1-SG | 6.43±0.10 | 0.30±0.01 | 9.69±0.48 | 1.76±0.44^ab^ | 158.05±6.59 | 16.06±0.88 | 1.72±0.07 | 20.55±0.87 | 11.95±0.29 |
| H1-BB | 6.19±0.07 | 0.30±0.01 | 11.35±1.01 | 2.47±0.78^a^ | 169.01±4.65 | 16.24±1.01 | 1.79±0.08 | 21.73±1.03 | 12.13±0.11 |
| H2-SG | 6.20±0.05 | 0.15±0.00 | 7.57±0.57 | 1.42±0.42^ab^ | 225.13±6.71 | 25.20±3.81 | 1.89±0.04 | 22.10±0.53 | 11.67±0.17 |
| H2-BB | 6.17±0.04 | 0.15±0.01 | 6.80±0.40 | 0.31±0.15^b^ | 221.09±8.77 | 19.98±1.39 | 1.79±0.08 | 21.26±0.85 | 11.93±0.17 |
| G-0N-SG | 6.49±0.20 | 0.24±0.01 | 14.44±2.00 | 0.80±0.14 | 211.52±1.68 | 20.59±0.59 | 1.79±0.07 | 21.43±0.07 | 12.00±0.45 |
| G-67N-SG | 6.36±0.08 | 0.24±0.00 | 10.54±0.89 | 1.54±0.24 | 204.90±11.62 | 18.91±1.00 | 1.80±0.01 | 20.96±0.38 | 11.67±0.23 |
| G-202N-SG | 6.23±0.15 | 0.26±0.00 | 9.98±0.61 | 3.13±0.62 | 194.83±2.38 | 19.55±1.75 | 2.04±0.08 | 23.79±1.19 | 11.65±0.13 |
| G-0N-BB | 6.25±0.06 | 0.25±0.01 | 10.86±0.84 | 0.21±0.14 | 225.96±1.84 | 19.56±1.49 | 1.79±0.06 | 21.55±0.87 | 12.01±0.26 |
| G-67N-BB | 6.26±0.06 | 0.24±0.00 | 11.21±0.62 | 0.87±0.21 | 204.99±9.87 | 17.57±0.97 | 1.73±0.09 | 21.23±1.54 | 12.22±0.28 |
| G-202N-BB | 6.09±0.03 | 0.23±0.00 | 9.10±0.37 | 2.27±0.88 | 181.77±2.51 | 16.45±1.36 | 1.77±0.04 | 21.38±1.04 | 12.09±0.31 |
| H1-0N-SG | 6.58±0.17 | 0.29±0.01 | 9.49±1.55 | 0.52±0.34 | 160.41±4.48 | 15.63±0.08 | 1.58±0.04 | 20.19±2.00 | 12.74±0.94 |
| H1-67N-SG | 6.50±0.05 | 0.31±0.01 | 9.51±0.82 | 1.49±0.35 | 150.67±17.47 | 16.25±2.58 | 1.71±0.08 | 20.12±0.86 | 11.80±0.07 |
| H1-202N-SG | 6.25±0.21 | 0.30±0.01 | 10.00±0.43 | 2.87±0.69 | 163.85±6.69 | 16.18±0.65 | 1.83±0.17 | 21.23±1.97 | 11.59±0.24 |
| H1-0N-BB | 6.33±0.05 | 0.30±0.01 | 9.28±1.77 | 0.77±0.75 | 174.97±9.88 | 14.93±2.03 | 1.61±0.10 | 19.34±1.08 | 12.03±0.25 |
| H1-67N-BB | 6.25±0.14 | 0.31±0.01 | 11.35±2.13 | 1.70±0.78 | 168.87±7.78 | 16.21±1.34 | 1.80±0.14 | 22.21±1.94 | 12.31±0.20 |
| H1-202N-BB | 5.99±0.11 | 0.29±0.01 | 13.43±0.62 | 4.93±1.16 | 163.18±8.19 | 17.58±2.13 | 1.96±0.16 | 23.64±1.77 | 12.05±0.13 |
| H2-0N-SG | 6.39±0.04 | 0.14±0.00 | 6.15±0.97 | 0.11±0.08 | 233.07±4.94 | 15.80±4.28 | 1.86±0.05 | 22.87±0.40 | 12.33±0.14 |
| H2-67N-SG | 6.19±0.05 | 0.16±0.01 | 8.09±0.41 | 1.14±0.27 | 227.73±18.46 | 24.75±1.08 | 1.99±0.06 | 23.02±0.66 | 11.55±0.20 |
| H2-202N-SG | 6.09±0.06 | 0.15±0.01 | 7.99±1.25 | 2.56±0.53 | 217.25±3.50 | 31.92±8.56 | 1.82±0.06 | 20.67±0.68 | 11.36±0.09 |
| H2-0N-BB | 6.24±0.03 | 0.16±0.01 | 6.66±0.87 | 0.02±0.00 | 246.63±7.31 | 19.10±3.83 | 1.68±0.16 | 19.78±1.57 | 11.78±0.18 |
| H2-67N-BB | 6.18±0.12 | 0.14±0.01 | 6.51±0.92 | 0.07±0.05 | 221.13±6.81 | 20.76±2.72 | 1.91±0.16 | 22.91±1.09 | 12.10±0.52 |
| H2-202N-BB | 6.08±0.04 | 0.15±0.01 | 7.22±0.41 | 0.85±0.25 | 195.52±13.07 | 20.10±0.65 | 1.77±0.13 | 21.10±1.56 | 11.92±0.12 |

^†^G = grass green up; H1 = initial grass harvest; H2 = second grass harvest; 0N = no N fertilization; 67N = 67 kg N ha^-1^ fertilization; 202N = 202 kg N ha^-1^ fertilization; SG = switchgrass; BB = big bluestem.

^‡^SWC = soil water content.

**Table S2.** Results of mixed model ANOVA (based on GLIMMIX procedure in SAS) testing effects of agricultural season, nitrogen fertilization rate, and grass species on the alpha-diversity metrics of *nifH* genes. F-values are reported.

| Alpha diversity | Chao1 estimator | Observed OTUs | Phylogenetic diversity | Shannon index | Pielou’s evenness |
| --- | --- | --- | --- | --- | --- |
| Season | **13.06***** | **15.39***** | **13.48***** | **3.93*** | 2.57 |
| Nitrogen | 2.80 | **8.55**** | **10.97***** | **11.77***** | **6.98**** |
| Grass | 0.02 | 0.77 | 0.21 | 4.16 | 2.46 |
| Season×Nitrogen | 1.66 | 2.39 | 2.52 | 0.58 | 0.37 |
| Season×Grass | 3.55 | 2.86 | 1.67 | 0.85 | 0.65 |
| Nitrogen×Grass | 0.07 | 0.10 | 1.41 | 1.39 | 1.52 |
| Season×Nitrogen×Grass | 1.76 | 1.63 | 2.02 | 1.08 | 1.44 |

Significance: * 0.01 < *p*-value ≤ 0.05; ** 0.001 *< p*-value ≤ 0.01; *** *p*-value ≤ 0.001.

**Table S3.** Results of pairwise PERMANOVA of diazotrophic community in three agricultural seasons and under different N fertilization rate and grass species. The *q*-value is the adjusted *p*-value with a Benjamini & Hochberg correction. The results were considered as significant when *q*-value ≤ 0.05.

| Treatment | Group | Pseudo-F | *p*-value | *q*-value |
| --- | --- | --- | --- | --- |
| Season | G^†^/H1 | 2.285 | 0.076 | 0.076 |
|  | G/H2 | 2.315 | 0.070 | 0.076 |
|  | H1/H2 | 7.550 | 0.001 | 0.003** |
| Nitrogen | 0N/67N | 1.224 | 0.282 | 0.282 |
|  | 0N/202N | 11.760 | 0.001 | 0.003** |
|  | 67N/202N | 4.738 | 0.007 | 0.011* |
| Grass | SG/BB | 3.486 | 0.016 | 0.016* |
| Nitrogen  ×  Grass | 0N-BB/0N-SG | 1.313 | 0.248 | 0.266 |
|  | 0N-BB/67N-BB | 0.898 | 0.426 | 0.426 |
|  | 0N-BB/67N-SG | 3.497 | 0.025 | 0.047* |
|  | 0N-BB/202N-BB | 4.874 | 0.001 | 0.004** |
|  | 0N-BB/202N-SG | 11.136 | 0.001 | 0.004** |
|  | 0N-SG/67N-BB | 1.470 | 0.176 | 0.203 |
|  | 0N-SG/67N-SG | 2.019 | 0.120 | 0.164 |
|  | 0N-SG/202N-BB | 3.720 | 0.008 | 0.020* |
|  | 0N-SG/202N-SG | 9.692 | 0.001 | 0.004** |
|  | 67N-BB/67N-SG | 3.761 | 0.021 | 0.045* |
|  | 67N-BB/202N-BB | 3.848 | 0.007 | 0.020* |
|  | 67N-BB/202N-SG | 9.416 | 0.001 | 0.004** |
|  | 67N-SG/202N-BB | 1.680 | 0.157 | 0.196 |
|  | 67N-SG/202N-SG | 3.058 | 0.048 | 0.080 |
|  | 202N-BB/202N-SG | 2.248 | 0.117 | 0.164 |
| Season  ×  Nitrogen | G_0N/G_67N | 1.199 | 0.270 | 0.360 |
|  | G_0N/G_202N | 4.525 | 0.012 | 0.039* |
|  | G_67N/G_202N | 1.149 | 0.326 | 0.419 |
|  | G_0N/H1_0N | 0.811 | 0.559 | 0.610 |
|  | G_0N/H2_0N | 1.097 | 0.361 | 0.448 |
|  | G_67N/H1_67N | 0.513 | 0.741 | 0.762 |
|  | G_67N/H2_67N | 2.170 | 0.058 | 0.110 |
|  | G_202N/H1_202N | 3.507 | 0.064 | 0.110 |
|  | G_202N/H2_202N | 0.747 | 0.548 | 0.610 |
|  | H1_0N/H1_67N | 0.941 | 0.447 | 0.519 |
|  | H1_0N/H1_202N | 6.231 | 0.028 | 0.068 |
|  | H1_67N/H1_202N | 2.340 | 0.088 | 0.144 |
|  | H1_0N/H2_0N | 2.335 | 0.048 | 0.102 |
|  | H1_67N/H2_67N | 3.687 | 0.013 | 0.039* |
|  | H1_202N/H2_202N | 5.316 | 0.020 | 0.055 |
|  | H2_0N/H2_67N | 0.380 | 0.938 | 0.938 |
|  | H2_0N/H2_202N | 4.694 | 0.006 | 0.031* |
|  | H2_67N/H2_202N | 4.592 | 0.007 | 0.032* |
| Season  ×  Grass | G_SG/G_BB | 1.577 | 0.181 | 0.265 |
|  | G_SG/H1_SG | 1.598 | 0.184 | 0.265 |
|  | G_SG/H2_SG | 1.181 | 0.313 | 0.361 |
|  | G_BB/H1_BB | 0.854 | 0.503 | 0.503 |
|  | G_BB/H2_BB | 1.508 | 0.194 | 0.265 |
|  | H1_SG/H1_BB | 0.993 | 0.339 | 0.363 |
|  | H1_SG/H2_SG | 4.576 | 0.019 | 0.078 |
|  | H1_BB/H2_BB | 3.468 | 0.006 | 0.045* |
|  | H2_SG/H2_BB | 2.055 | 0.072 | 0.135 |

Significance: *0.01< *q*-value ≤ 0.05; **0.001 < *q*-value ≤ 0.01; *** *q*-value ≤ 0.001.

^†^G = grass green up; H1 = initial grass harvest; H2 = second grass harvest; 0N = no N fertilization; 67N = 67 kg N ha^-1^ fertilization; 202N = 202 kg N ha^-1^ fertilization; SG = switchgrass; BB = big bluestem.

**Table S4.** Results from indicator species analysis aiming at identifying *nifH* OTUs typical of different combination of N fertilization rate and grass species or groups of the combinations. The classification of OTUs at phylum, class and order level, as well as the specificity value (A-value), fidelity value (B-value), indicator value index (stat-value) and the significance (*p*-value) of the test are reported.

| **0N-SG**^†^ |  |  |  |  |  |  |  |
| --- | --- | --- | --- | --- | --- | --- | --- |
| *OTU* | *Phylum* | *Class* | *Order* | *A* | *B* | *stat* | *p. value* |
| OTU169 | Proteobacteria | Gammaproteobacteria | Pseudomonadales | 0.885 | 0.667 | 0.768 | 0.002** |
| OTU207 | Proteobacteria | Alphaproteobacteria | Rhizobiales | 0.702 | 0.778 | 0.739 | 0.009** |
| **0N-BB** |  |  |  |  |  |  |  |
| *OTU* | *Phylum* | *Class* | *Order* | *A* | *B* | *stat* | *p. value* |
| OTU198 | Proteobacteria | Alphaproteobacteria | Rhizobiales | 0.936 | 0.375 | 0.593 | 0.048* |
| **67N-BB** |  |  |  |  |  |  |  |
| *OTU* | *Phylum* | *Class* | *Order* | *A* | *B* | *stat* | *p. value* |
| OTU215 | Proteobacteria | Alphaproteobacteria | Rhizobiales | 0.931 | 0.857 | 0.893 | 0.001*** |
| **202N-SG & 202N-BB** | | |  |  |  |  |  |
| *OTU* | *Phylum* | *Class* | *Order* | *A* | *B* | *stat* | *p. value* |
| OTU185 | Proteobacteria | Alphaproteobacteria | Rhizobiales | 1 | 0.588 | 0.767 | 0.003** |
| **0N-BB & 67N-BB** | |  |  |  |  |  |  |
| *OTU* | *Phylum* | *Class* | *Order* | *A* | *B* | *stat* | *p. value* |
| OTU144 | Proteobacteria | Alphaproteobacteria | Rhizobiales | 0.7561 | 1 | 0.87 | 0.009** |
| OTU122 | Verrucomicrobia | Opitutae | Opitutales | 0.840 | 0.867 | 0.853 | 0.001*** |
| OTU165 | Proteobacteria | Alphaproteobacteria | Rhodospirillales | 0.9285 | 0.6667 | 0.787 | 0.001*** |
| OTU96 | Proteobacteria | Alphaproteobacteria | Rhizobiales | 0.988 | 0.533 | 0.726 | 0.01** |
| OTU138 | Proteobacteria | Betaproteobacteria | Rhodocyclales | 0.951 | 0.533 | 0.712 | 0.019* |
| OTU186 | Proteobacteria | Alphaproteobacteria | Rhizobiales | 0.947 | 0.533 | 0.711 | 0.007** |
| **0N-SG & 67N-SG & 0N-BB** | | |  |  |  |  |  |
| *OTU* | *Phylum* | *Class* | *Order* | *A* | *B* | *stat* | *p. value* |
| OTU77 | Proteobacteria | Alphaproteobacteria | Rhizobiales | 0.883 | 0.962 | 0.921 | 0.001*** |
| OTU112 | Proteobacteria | Alphaproteobacteria | Rhizobiales | 0.906 | 0.923 | 0.914 | 0.001*** |
| OTU95 | Proteobacteria | Alphaproteobacteria | Rhodospirillales | 0.918 | 0.885 | 0.901 | 0.001*** |
| OTU12 | Proteobacteria | Alphaproteobacteria | Rhizobiales | 0.881 | 0.885 | 0.883 | 0.001*** |
| OTU115 | Proteobacteria | Alphaproteobacteria | Rhizobiales | 0.931 | 0.808 | 0.867 | 0.001*** |
| **0N-SG & 0N-BB & 67N-BB** | | |  |  |  |  |  |
| *OTU* | *Phylum* | *Class* | *Order* | *A* | *B* | *stat* | *p. value* |
| OTU132 | Proteobacteria | Alphaproteobacteria | Rhizobiales | 0.800 | 1 | 0.894 | 0.016* |
| OTU87 | Proteobacteria | Gammaproteobacteria | Chromatiales | 0.895 | 0.875 | 0.885 | 0.001*** |
| OTU116 | Proteobacteria | Alphaproteobacteria | Rhizobiales | 0.912 | 0.833 | 0.872 | 0.002** |
| OTU204 | Proteobacteria | Betaproteobacteria | Rhodocyclales | 0.9101 | 0.7083 | 0.803 | 0.004** |
| OTU78 | Proteobacteria | Alphaproteobacteria | Rhizobiales | 0.943 | 0.667 | 0.793 | 0.004** |
| **67N-SG & 0N-BB & 67N-BB** | | |  |  |  |  |  |
| *OTU* | *Phylum* | *Class* | *Order* | *A* | *B* | *stat* | *p. value* |
| OTU150 | Proteobacteria | Alphaproteobacteria | Rhizobiales | 0.959 | 0.792 | 0.871 | 0.002** |
| **202N-SG & 67N-BB & 202N-BB** | | | |  |  |  |  |
| *OTU* | *Phylum* | *Class* | *Order* | *A* | *B* | *stat* | *p. value* |
| OTU208 | Proteobacteria | Alphaproteobacteria | Rhizobiales | 0.884 | 0.958 | 0.92 | 0.001*** |
| OTU156 | Proteobacteria | Alphaproteobacteria | Rhizobiales | 0.885 | 0.917 | 0.901 | 0.001*** |
| OTU161 | Proteobacteria | Alphaproteobacteria | Rhizobiales | 0.913 | 0.542 | 0.703 | 0.017* |
| **0N-BB & 67N-BB & 202N-BB** | | |  |  |  |  |  |
| *OTU* | *Phylum* | *Class* | *Order* | *A* | *B* | *stat* | *p. value* |
| OTU58 | Proteobacteria | Alphaproteobacteria | Rhizobiales | 0.905 | 0.917 | 0.911 | 0.002** |
| OTU93 | Proteobacteria | Alphaproteobacteria | Rhizobiales | 0.855 | 0.750 | 0.801 | 0.006** |
| **0N-SG & 67N-SG & 202N-SG & 0N-BB** | | | |  |  |  |  |
| *OTU* | *Phylum* | *Class* | *Order* | *A* | *B* | *stat* | *p. value* |
| OTU120 | Proteobacteria | Alphaproteobacteria | Sphingomonadales | 0.957 | 0.824 | 0.888 | 0.002** |
| OTU230 | Proteobacteria | Alphaproteobacteria | Rhizobiales | 0.950 | 0.794 | 0.869 | 0.001*** |
| OTU134 | Proteobacteria | Alphaproteobacteria | Rhizobiales | 0.946 | 0.794 | 0.867 | 0.002** |
| **0N-SG & 67N-SG & 202N-SG & 202N-BB** | | | | |  |  |  |
| *OTU* | *Phylum* | *Class* | *Order* | *A* | *B* | *stat* | *p. value* |
| OTU24 | Proteobacteria | Alphaproteobacteria | Rhizobiales | 0.933 | 0.914 | 0.924 | 0.016* |
| OTU234 | Proteobacteria | Alphaproteobacteria | Rhizobiales | 0.956 | 0.771 | 0.859 | 0.002** |
| **0N-SG & 67N-SG & 0N-BB & 67N-BB** | | | |  |  |  |  |
| *OTU* | *Phylum* | *Class* | *Order* | *A* | *B* | *stat* | *p. value* |
| OTU49 | Unclassified | Unclassified | Unclassified | 0.970 | 1 | 0.985 | 0.001*** |
| OTU111 | Proteobacteria | Alphaproteobacteria | Rhizobiales | 0.947 | 0.879 | 0.912 | 0.001*** |
| OTU19 | Proteobacteria | Alphaproteobacteria | Rhizobiales | 0.9824 | 0.8182 | 0.897 | 0.006** |
| OTU41 | Proteobacteria | Alphaproteobacteria | Rhizobiales | 0.953 | 0.818 | 0.883 | 0.007** |
| OTU34 | Proteobacteria | Alphaproteobacteria | Rhizobiales | 0.968 | 0.788 | 0.873 | 0.013* |
| OTU69 | Cyanobacteria | Cyanophyceae | Nostocales | 1 | 0.697 | 0.835 | 0.002** |
| OTU130 | Proteobacteria | Alphaproteobacteria | Rhizobiales | 0.969 | 0.697 | 0.822 | 0.002** |
| OTU106 | Proteobacteria | Alphaproteobacteria | Rhizobiales | 0.994 | 0.636 | 0.795 | 0.007** |
| OTU211 | Cyanobacteria | Cyanophyceae | Nostocales | 0.963 | 0.576 | 0.744 | 0.03* |
| OTU149 | Proteobacteria | Alphaproteobacteria | Rhizobiales | 0.963 | 0.515 | 0.704 | 0.041* |
| **0N-SG & 202N-SG & 67N-BB & 202N-BB** | | | | |  |  |  |
| *OTU* | *Phylum* | *Class* | *Order* | *A* | *B* | *stat* | *p. value* |
| OTU108 | Proteobacteria | Alphaproteobacteria | Rhizobiales | 0.957 | 0.939 | 0.948 | 0.001*** |
| OTU135 | Chlorobi | Chlorobia | Chlorobiales | 0.952 | 0.818 | 0.882 | 0.001*** |
| OTU159 | Proteobacteria | Alphaproteobacteria | Rhizobiales | 0.900 | 0.758 | 0.826 | 0.01** |
| **0N-SG & 0N-BB & 67N-BB & 202N-BB** | | | |  |  |  |  |
| *OTU* | *Phylum* | *Class* | *Order* | *A* | *B* | *stat* | *p. value* |
| OTU94 | Proteobacteria | Deltaproteobacteria | Desulfovibrionales | 0.935 | 0.909 | 0.922 | 0.004** |
| OTU236 | Proteobacteria | Deltaproteobacteria | Desulfuromonadales | 0.983 | 0.667 | 0.809 | 0.001*** |
| **202N-SG & 0N-BB & 67N-BB & 202N-BB** | | | | |  |  |  |
| *OTU* | *Phylum* | *Class* | *Order* | *A* | *B* | *stat* | *p. value* |
| OTU197 | Proteobacteria | Alphaproteobacteria | Rhizobiales | 0.923 | 0.594 | 0.74 | 0.045* |
| **0N-SG & 67N-SG & 202N-SG & 0N-BB & 67N-BB** | | | | |  |  |  |
| *OTU* | *Phylum* | *Class* | *Order* | *A* | *B* | *stat* | *p. value* |
| OTU35 | Proteobacteria | Alphaproteobacteria | Rhizobiales | 0.976 | 0.951 | 0.963 | 0.01** |
| OTU137 | Proteobacteria | Alphaproteobacteria | Rhizobiales | 0.988 | 0.854 | 0.918 | 0.001*** |
| OTU154 | Proteobacteria | Alphaproteobacteria | Sphingomonadales | 0.964 | 0.829 | 0.894 | 0.014* |
| **0N-SG & 67N-SG & 202N-SG & 0N-BB & 202N-BB** | | | | |  |  |  |
| *OTU* | *Phylum* | *Class* | *Order* | *A* | *B* | *stat* | *p. value* |
| OTU16 | Proteobacteria | Alphaproteobacteria | Sphingomonadales | 0.966 | 0.977 | 0.971 | 0.003** |
| OTU141 | Proteobacteria | Alphaproteobacteria | Sphingomonadales | 0.924 | 0.954 | 0.938 | 0.01** |
| **0N-SG & 67N-SG & 202N-SG & 67N-BB & 202N-BB** | | | | | |  |  |
| *OTU* | *Phylum* | *Class* | *Order* | *A* | *B* | *stat* | *p. value* |
| OTU40 | Proteobacteria | Betaproteobacteria | Rhodocyclales | 0.971 | 0.976 | 0.973 | 0.002** |
| OTU126 | Proteobacteria | Betaproteobacteria | Burkholderiales | 0.961 | 0.905 | 0.932 | 0.007** |
| OTU128 | Proteobacteria | Alphaproteobacteria | Rhizobiales | 0.965 | 0.881 | 0.922 | 0.016* |
| OTU125 | Proteobacteria | Alphaproteobacteria | Rhizobiales | 0.983 | 0.762 | 0.865 | 0.024* |
| OTU117 | Proteobacteria | Alphaproteobacteria | Rhizobiales | 0.993 | 0.738 | 0.856 | 0.01** |
| **0N-SG & 67N-SG & 0N-BB & 67N-BB & 202N-BB** | | | | |  |  |  |
| *OTU* | *Phylum* | *Class* | *Order* | *A* | *B* | *stat* | *p. value* |
| OTU32 | Proteobacteria | Alphaproteobacteria | Rhizobiales | 0.979 | 0.976 | 0.978 | 0.002** |
| OTU36 | Proteobacteria | Alphaproteobacteria | Rhizobiales | 0.991 | 0.952 | 0.971 | 0.002** |
| OTU101 | Proteobacteria | Alphaproteobacteria | Rhizobiales | 0.985 | 0.952 | 0.968 | 0.002** |
| OTU48 | Proteobacteria | Gammaproteobacteria | Pseudomonadales | 0.978 | 0.952 | 0.965 | 0.006** |
| OTU109 | Proteobacteria | Alphaproteobacteria | Rhizobiales | 0.959 | 0.857 | 0.907 | 0.044* |
| OTU148 | Proteobacteria | Gammaproteobacteria | Pseudomonadales | 0.984 | 0.833 | 0.906 | 0.003** |
| OTU140 | Proteobacteria | Alphaproteobacteria | Rhizobiales | 0.956 | 0.810 | 0.88 | 0.028* |
| OTU114 | Proteobacteria | Alphaproteobacteria | Rhodospirillales | 0.993 | 0.762 | 0.87 | 0.017* |
| OTU168 | Proteobacteria | Alphaproteobacteria | Rhizobiales | 0.963 | 0.762 | 0.857 | 0.043* |
| OTU146 | Proteobacteria | Alphaproteobacteria | Rhodospirillales | 0.985 | 0.714 | 0.839 | 0.025* |
| OTU86 | Proteobacteria | Alphaproteobacteria | Rhizobiales | 0.997 | 0.691 | 0.83 | 0.019* |
| **0N-SG & 202N-SG & 0N-BB & 67N-BB & 202N-BB** | | | | |  |  |  |
| *OTU* | *Phylum* | *Class* | *Order* | *A* | *B* | *stat* | *p. value* |
| OTU110 | Proteobacteria | Alphaproteobacteria | Rhizobiales | 0.975 | 0.878 | 0.925 | 0.002** |
| OTU164 | Proteobacteria | Alphaproteobacteria | Rhizobiales | 0.979 | 0.805 | 0.888 | 0.02* |

Significance level: * 0.01 < *p*-value ≤ 0.05; ** 0.001 < *p*-value ≤ 0.01; *** *p*-value ≤ 0.001.

^†^0N = no N fertilization; 67N = 67 kg N ha^-1^ fertilization; 202N = 202 kg N ha^-1^ fertilization; SG = switchgrass; BB = big bluestem.

**Table S5.** Spearman correlation coefficients among abundances of *nifH* genes and transcripts, alpha-diversity metrics of *nifH* genes, and soil properties.

|  | pH | SWC^†^ | NH_4_^+^-N | NO_3_^-^-N | DOC | DON | Total C | Total N | C: N ratio | *nifH* gene copies gdw^-1^ | *nifH* transcript copies gdw^-1^ |
| --- | --- | --- | --- | --- | --- | --- | --- | --- | --- | --- | --- |
| *nifH* gene copies gdw^-1^ | -0.043 | -.781** | -.510** | -.387** | .728** | .510** | -0.127 | 0.039 | -.347* | 1 | -.349* |
| *nifH* transcript copies gdw^-1^ | .423** | .590** | .320* | 0.002 | -.280* | -0.219 | -0.092 | -0.076 | 0.011 | -.349* | 1 |
| Chao1 estimator | 0.130 | -.404** | -.339* | -.332* | .425** | 0.187 | -0.243 | -0.158 | -0.118 | .690** | -0.041 |
| Observed OTUs | 0.144 | -.324* | -0.265 | -.436** | .403** | 0.080 | -0.180 | -0.173 | 0.049 | .614** | 0.038 |
| Phylogenetic  diversity | 0.198 | -.355* | -0.246 | -.414** | .377** | 0.060 | -0.201 | -0.172 | 0.019 | .600** | -0.005 |
| Shannon index | 0.046 | 0.041 | 0.196 | -.359* | 0.122 | -0.166 | -0.039 | -0.168 | .422** | -0.093 | 0.235 |
| Pielou’s  evenness | 0.014 | 0.221 | .320* | -0.180 | -0.119 | -0.240 | 0.025 | -0.125 | .454** | -.381** | 0.243 |

** Correlation is significant at the 0.01 level (2-tailed).

* Correlation is significant at the 0.05 level (2-tailed).

^†^SWC = soil water content; DOC = dissolved organic C; DON = dissolved organic N.

**Table S6.** Specific standardized regression weight (path coefficient) for each pathway in the structural equation modeling for diazotrophs and soil properties.

| From | To | Path  coefficient | From | To | Path  coefficient |
| --- | --- | --- | --- | --- | --- |
| Grass species | Observed OTUs | 0.104 | Diazotrophic community | TN | -0.130 |
| Season | SWC | -0.600 | SWC | DOC | -0.618 |
| N fertilization | Nitrate | 0.689 | SWC | *nifH* gene abundance | -0.334 |
| SWC^†^ | Nitrate | 0.354 | Season | Ammonium | -0.325 |
| Grass species | Nitrate | -0.224 | SWC | Ammonium | 0.404 |
| SWC | DON | -0.604 | Season | DOC | -0.205 |
| Observed OTUs | Shannon | 0.481 | N fertilization | DOC | -0.297 |
| Nitrate | DON | 0.375 | Diazotrophic community | Rhodocyclales | 0.463 |
| SWC | Diazotrophic community | 0.677 | SWC | *nifH* transcript abundance | 0.602 |
| Grass species | Diazotrophic community | 0.233 | Observed OTUs | *nifH* gene abundance | 0.427 |
| Grass species | pH | -0.443 | TN | DOC | 0.370 |
| Observed OTUs | Chao1 | 1.044 | Diazotrophic community | Rhizobiales | 0.821 |
| Nitrate | Chao1 | 0.109 | Diazotrophic community | Sphingomonadales | -0.732 |
| Season | Chao1 | 0.078 | Diazotrophic community | Rhodospirillales | -0.931 |
| DON | pH | -0.303 | DON | *nifH* gene abundance | 0.154 |
| TOC | pH | -0.243 | Diazotrophic community | *nifH* gene abundance | -0.364 |
| Nitrate | TN | 0.147 | Evenness | *nifH* gene abundance | -0.177 |
| TOC | Chao1 | -0.081 | Chao1 | DOC | 0.301 |
| Shannon | Chao1 | -0.190 | Grass species | DOC | 0.149 |
| Evenness | TN | -0.107 | Evenness | Rhodocyclales | 0.321 |
| Nitrate | pH | -0.369 | pH | Rhodocyclales | -0.248 |

^†^SWC, soil water content; DOC, dissolved organic C; DON, dissolved organic N; TOC, total organic C; TN, total N.

**Table S7.** Standardized total effects from the structural equation model relating agricultural season, grass species, N fertilization rate, and soil physicochemical properties on diazotrophic microbial abundance, functional activity, diversity, and major community composition.

| **Factors** | **Grass**  **species** | **Nitrogen**  **fertilization** | **Season** | **SWC^†^** | **nitrate** | **TOC** | **DON** | **pH** |
| --- | --- | --- | --- | --- | --- | --- | --- | --- |
| **Observed OTUs** | 0.104 | 0.000 | 0.000 | 0.000 | 0.000 | 0.000 | 0.000 | 0.000 |
| **Shannon index** | 0.050 | 0.000 | 0.000 | 0.000 | 0.000 | 0.000 | 0.000 | 0.000 |
| **Chao1 index** | 0.074 | 0.075 | 0.055 | 0.039 | 0.109 | -0.081 | 0.000 | 0.000 |
| **Diazotrophic community** | 0.207 | 0.000 | -0.406 | 0.677 | 0.000 | 0.000 | 0.000 | 0.000 |
| **Rhodospirillales** | -0.193 | 0.000 | 0.378 | -0.630 | 0.000 | 0.000 | 0.000 | 0.000 |
| **Sphingomonadales** | -0.151 | 0.000 | 0.297 | -0.495 | 0.000 | 0.000 | 0.000 | 0.000 |
| **Rhizobiales** | 0.170 | 0.000 | -0.333 | 0.555 | 0.000 | 0.000 | 0.000 | 0.000 |
| **Rhodocyclales** | 0.179 | 0.082 | -0.186 | 0.310 | 0.120 | 0.060 | 0.075 | -0.248 |
| ***nifH* transcript abundance** | -0.023 | 0.000 | -0.361 | 0.602 | 0.000 | 0.000 | 0.000 | 0.000 |
| ***nifH* gene abundance** | -0.028 | 0.040 | 0.392 | -0.654 | 0.058 | 0.000 | 0.154 | 0.000 |

^†^SWC, soil water content; TOC, total organic C; DON, dissolved organic N.

**Table S8.** Spearman correlation coefficients between alpha-diversity of diazotrophic microbial community and *nifH* gene and transcript abundances [log (copies gdw^-1^)] within different treatment combinations.

|  | Treatment | Group | Chao1 estimator | Observed OTUs | Phylogenetic diversity | Shannon index |
| --- | --- | --- | --- | --- | --- | --- |
| *nifH* gene abundance | Overall | | .690** | .614** | .600** | -0.093 |
|  | Season | G^†^ | .645** | .613** | .662** | -.605* |
|  |  | H1 | .632* | .661** | .668** | .136 |
|  |  | H2 | .659** | .527* | .352 | -.051 |
|  | Nitrogen | 0N | .662** | .529* | .554* | -.400 |
|  |  | 67N | .803** | .794** | .753** | -.068 |
|  |  | 202N | .699** | .574* | .554* | .088 |
|  | Grass | SG | .770** | .724** | .658** | -.022 |
|  |  | BB | .439* | .379 | .445* | -.189 |
| *nifH* transcript abundance | Overall | | -0.041 | 0.038 | -0.005 | 0.235 |
|  | Season | G^†^ | .186 | .350 | .201 | .407 |
|  |  | H1 | .518* | .529* | .554* | .318 |
|  |  | H2 | .051 | .155 | .133 | .209 |
|  | Nitrogen | 0N | -.228 | .002 | -.059 | .120 |
|  |  | 67N | -.406 | -.338 | -.450 | .018 |
|  |  | 202N | .199 | .176 | .135 | .086 |
|  | Grass | SG | .052 | .124 | .068 | .007 |
|  |  | BB | -.203 | -.079 | -.166 | .552** |

** Correlation is significant at the 0.01 level (2-tailed).

* Correlation is significant at the 0.05 level (2-tailed).

^†^G = grass green up; H1 = initial grass harvest; H2 = second grass harvest; 0N = no N fertilization; 67N = 67 kg N ha^-1^ fertilization; 202N = 202 kg N ha^-1^ fertilization; SG = switchgrass; BB = big bluestem.

**Table S9.** Four abundant *nifH* OTUs that significantly correlated to *nifH* gene and transcript abundances [log (copies gdw^-1^)]. Spearman correlation coefficients between relative abundance of these four OTUs and *nifH* gene copy numbers were shown.

| Spearman correlation | OTU1 | OTU5 | | OTU6 | | OTU18 | |
| --- | --- | --- | --- | --- | --- | --- | --- |
| *nifH* gene abundance | .683** | | .722** | | .724** | | .519* |
| *nifH* transcript abundance | -.389** | | -.246 | | -.306* | | .115 |

**. Correlation is significant at the 0.01 level (2-tailed).

*. Correlation is significant at the 0.05 level (2-tailed).

## Supplementary Figures


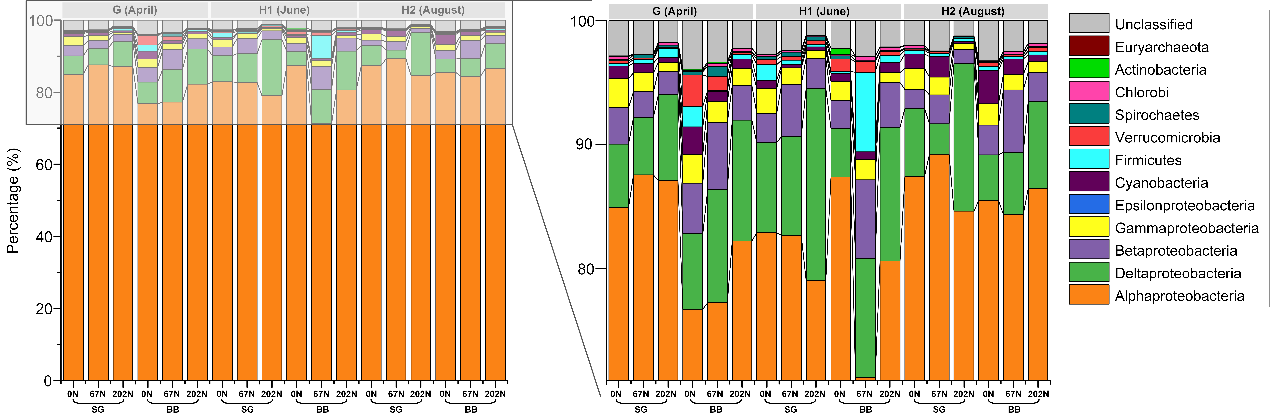


**Figure S1.** Composition of *nifH* taxa in different treatment combinations in different sampling times. G, grass green up; H1, initial grass harvest; H2, second grass harvest; 0N, no N fertilization; 67N, 67 kg N ha^-1^ fertilization; 202N, 202 kg N ha^-1^ fertilization; SG, switchgrass; BB, big bluestem.
